# Crosstalk of DNA Methylation Triggered by Pathogen in Poplars With Different Resistances

**DOI:** 10.3389/fmicb.2021.750089

**Published:** 2021-12-28

**Authors:** Dandan Xiao, Ke Zhou, Xiaoqian Yang, Yuzhang Yang, Yudie Ma, Yanwei Wang

**Affiliations:** ^1^National Engineering Laboratory for Tree Breeding, College of Biological Sciences and Biotechnology, Beijing Forestry University, Beijing, China; ^2^Key Laboratory of Genetics and Breeding in Forest Trees and Ornamental Plants, Ministry of Education, College of Biological Sciences and Biotechnology, Beijing Forestry University, Beijing, China; ^3^The Tree and Ornamental Plant Breeding and Biotechnology Laboratory of National Forestry and Grassland Administration, College of Biological Sciences and Biotechnology, Beijing Forestry University, Beijing, China; ^4^School of Landscape Architecture, Chengdu Agricultural College, Chengdu, China

**Keywords:** DNA methylation, poplar, *Lonsdalea populi*, infection, resistance

## Abstract

DNA methylation plays crucial roles in responses to environmental stimuli. Modification of DNA methylation during development and abiotic stress responses has been confirmed in increasing numbers of plants, mainly annual plants. However, the epigenetic regulation mechanism underlying the immune response to pathogens remains largely unknown in plants, especially trees. To investigate whether DNA methylation is involved in the response to infection process or is related to the resistance differences among poplars, we performed comprehensive whole-genome bisulfite sequencing of the infected stem of the susceptible type *Populus* × *euramerican* ‘74/76’ and resistant type *Populus tomentosa* ‘henan’ upon *Lonsdalea populi* infection. The results revealed that DNA methylation changed dynamically in poplars during the infection process with a remarkable decrease seen in the DNA methylation ratio. Intriguingly, the resistant *P. tomentosa* ‘henan’ had a much lower basal DNA methylation ratio than the susceptible *P.* × *euramerican* ‘74/76’. Compared to mock-inoculation, both poplar types underwent post-inoculation CHH hypomethylation; however, significant decreases in mC and mCHH proportions were found in resistant poplar. In addition, most differentially CHH-hypomethylated regions were distributed in repeat and promoter regions. Based on comparison of DNA methylation modification with the expression profiles of genes, DNA methylation occurred in resistance genes, pathogenesis-related genes, and phytohormone genes in poplars during pathogen infection. Additionally, transcript levels of genes encoding methylation-related enzymes changed during pathogen infection. Interestingly, small-regulator miRNAs were subject to DNA methylation in poplars experiencing pathogen infection. This investigation highlights the critical role of DNA methylation in the poplar immune response to pathogen infection and provides new insights into epigenetic regulation in perennial plants in response to biotic stress.

## Introduction

Epigenetic mechanisms, especially DNA methylation, are conserved and associated with gene silencing, chromatin remodeling, histone modification, and epigenetic variations induced by the environment (Law and Jacobsen, 2010; Shipony et al., 2014; Tirnaz and Batley, 2019). Moreover, DNA methylation occurs throughout the whole life of the plant, playing a role in adaptation to stress conditions, genome management, and developmental regulation (Sherman and Talbert, 2002; Meng et al., 2003; Feng et al., 2014; Elhamamsy, 2016). In *Arabidopsis*, research results support a model whereby DNA methylation imparts persistent control over some defense genes under non-stressful conditions, but can shift dynamically to modulate gene expression in response to environmental stimuli (Dowen et al., 2012). In plants, all three types of cytosine DNA methylation (CG, CHG, and CHH) are catalyzed by DOMAINS REARRANGED METHYLTRA NSFERASE2 (DRM2), but with differing maintenance pathways. Symmetrical CG and CHG DNA methylation grouping is maintained by METHYLTRANSFER ASE1 (MET1) and CHROMOMETHYLASE3 (CMT3), respectively, while asymmetric CHH methylation is maintained by DRM2 (Law and Jacobsen, 2010; Zhang et al., 2018).

DNA methylation in plants changes in response to diverse abiotic stress conditions, including heat, cold, drought, high salinity, osmotic stress, ultraviolet radiation, soil nutrient deficiency, laser irradiation, metal stress, anoxia and re-oxygenation, pesticides, and climate change. These changes have been investigated thoroughly in a wide range of plants including *Arabidopsis*, cotton, winter wheat, rice, *Brassica rapa*, *Isoetes sinensis* Palmer, *Camellia sinensis*, and poplar (Sherman and Talbert, 2002; Song et al., 2016; Liu et al., 2017; Zheng et al., 2017; Zhang et al., 2018, 2019; Ding et al., 2019; Van Dooren et al., 2020; Zhu et al., 2020). Recently, reports of DNA methylation modification in plants in response to biotic stressors have increased. Biotic stressors include the effects of bacteria, fungi, and viruses that impact the normal growth and development of plants. DNA methylation has been reported to play a crucial role in plant resistance to biotic stress (Hewezi et al., 2017). For example, the level of DNA methylation in tobacco changed in response to external tobacco mosaic virus (TMV) infection, and this change was closely related to the activation of stress-responsive genes (Wada et al., 2004). Dynamic DNA methylation had been suggested to represent a regulatory layer in the complex mechanism of plant immunity, which may be exploited to improve disease resistance in common wheat (Geng et al., 2019). Additionally, DNA demethylation was presumed to be involved in the immune activity of *Arabidopsis* against microbial pathogens (Pavet et al., 2006). Together, these reports demonstrate that changes in DNA methylation in response to various biotic stressors are common in plants. However, these response patterns may differ among plant species, or in response to infection with different pathogens. In grape berries, melatonin treatment increased plant disease resistance and flavonoid biosynthesis by decreasing the methylation levels (MLs) of gene promoters (Gao et al., 2020). Stable methylation was observed in *Populus simonii* under cold, osmotic, heat, and salt stress, based on analysis of the methylome and gene expression (Song et al., 2016). Single-base-resolution methylomes of *Populus trichocarpa* showed a significant increase in cytosine methylation after drought treatment (Liang et al., 2014). These investigations demonstrated that DNA methylation plays important roles in perennial plant responses to abiotic stress. However, whether stable global DNA methylation is involved in the immune response to biotic stress in poplar, a model woody perennial plant, remains unknown.

*Lonsdalea* canker, caused by *Lonsdalea populi*, was first observed in poplars of *Populus* × *euramericana* ‘74/76’ and *P.* × *euramericana* ‘Zhonglin 46’ in both Henan and Shandong provinces of China in 2006 (Li et al., 2014). Large numbers of poplars in China and Hungary had been affected by *Lonsdalea* canker (Li and He, 2019). Several studies of this disease have been conducted on poplars and have identified the pathogen types, molecular basis of the pathogenesis of *L*. *populi*, tolerance/resistance of different poplar species, salicylic acid and jasmonic acid signal transduction pathways, and transcriptomic analysis of poplars infected with *L*. *populi* (Li et al., 2014; Liu Z. et al., 2015; Hou et al., 2016; Yang et al., 2018). Previous research has focused primarily on the bacterium itself, or on the plant immune response or poplar transcriptomes. Poplar species differing in survivability to *L*. *populi* infection have been investigated and validated, making it possible and necessary to explore the molecular mechanism of resistant and susceptible poplars showing different defense responses to *L*. *populi*. However, few reports have addressed whether the molecular mechanism of DNA methylation plays an important role in the immune responses of various poplar species.

Given increasing evidences for the involvement of DNA methylation in plant responses to biotic stress, as well as of a role of DNA methylation in regulating gene expression and genomic stability (Tirnaz and Batley, 2019), we hypothesized that DNA methylation changed in poplars during pathogen infection and may have important effects on the expression of biotic-stress-responsive genes. Here, the role of DNA methylation in the modification between two poplar species with different resistance levels and *L*. *populi* was comprehensively investigated at the genome-wide level. We found that DNA methylation changed dynamically during the inoculation process and showed similar change trends between the susceptible and resistant poplar types investigated, albeit with different DNA MLs. Additionally, the DNA methylation changes might be involved in differential expression of resistance (*R*) genes, pathogenesis-related (*PR*) genes, and phytohormone genes in poplar species. This investigation provides a new insight into the role of DNA methylation in the immune response upon infection of trees with bacterial pathogens, which could be potentially used in endowing resistance of perennial plants to pathogen infection.

## Materials and Methods

### Plant Growth and Treatment

Two-year-old *P. × euramericana* ‘74/76’ and *Populus tomentosa* ‘henan’ plantlets were grown under normal field conditions in the nursery of the Puyang Academy of Forestry (114° 87′ E, 35° 81′ N) in Henan Province, China. The plantlets were inoculated with *L*. *populi* strain N-5-1 (provided by Laboratory of Forest Pathology, Beijing Forestry University) or sterile water as a mock inoculation, as described previously (Hou et al., 2016).

Bark, including cambium, of the inoculated and mock inoculated poplar stems was sampled at 6 days post-inoculation (dpi) for both poplar types, which differed in resistance levels, and *P*. × *euramericana* trees were further sampled at 2 and 4 dpi for exploration of the dynamic response to infection of *L*. *populi*. Three biological replicates were conducted for each condition. Samples were immediately frozen in liquid nitrogen and stored at −80°C.

### DNA Extraction, Whole-Genome Bisulfite Sequencing and Library Preparation

Genomic DNA was extracted using a combination of cetyltrimethylammonium bromide (CTAB) and sodium dodecyl sulfate (SDS; Zhao et al., 2016). DNA purity and concentration were checked using the Nano Photometer^®^ spectrophotometer (Implen, Westlake Village, CA, United States). Genomic DNA degradation and contamination were monitored using 1% agarose gels.

The genomic and lambda DNA were fragmented via sonication to 200–300 bp with a Covaris S220 sonicator, followed by end repair and adenylation. Cytosine-methylated barcodes were ligated to the sonicated DNA. Then, these DNA fragments were treated with bisulfite using Accel-NGS^®^ Methyl-Seq DNA Library Kit in accordance with the manufacturer’s instructions. Library concentration was quantified with a Qubit^®^ 2.0 flurometer (Life Technologies, Carlsbad, CA, United States) and quantitative PCR, and the insert size was assessed with the Bioanalyzer 2100 system (Agilent, Santa Clara, CA, United States). The libraries were sequenced on a NovaSeq6000 sequencer (Novogene Co., Ltd., Beijing, China).

### Bioinformatic Mapping of Reads to the Reference Genome

Bismark software (v.0.16.3) (Krueger and Andrews, 2011) was used for alignment of bisulfite-treated reads to poplar genome v3.0^1^ with parameters set as –score_min L, 0, -0.2, -X 700 –dovetail. The reference genome was first transformed into a bisulfite-converted version (C-to-T and G-to-A) and then indexed using bowtie2 (v.2.2.5) (Langmead and Salzberg, 2012).

Sequence reads were transformed into fully bisulfite-converted versions before alignment to similarly converted versions of the genome in a directional manner. Sequence reads that produced a unique best hit from the two alignment processes (original top and bottom strand) were compared to the normal genomic sequence, and the methylation state of all cytosine positions in the read was inferred. Reads aligned to the same regions of the genome were regarded as duplicates. The sequencing depth and coverage were summarized using deduplicated reads.

### Estimation of Methylation Level

To identify methylation sites, we summed the methylated cytokines (mC) as a binomial (Bin) random variable with a methylation rate (r), which was calculated as follows: mC ∼ Bln (mC + umC*r). The sum of methylated and unmethylated read counts in each window was calculated. Bisulphite conversion efficiency was calculated based on lambda DNA genome. The reliability of methylation site level data was evaluated with a binomial distribution test, and thresholds were set to precisely determine the methylation sites, as follows: the sequencing depth ≥5; Q-value ≤ 0.01; and conversion rate ≥99%. The ML for each window or C site indicated the fraction of methylated Cs, and was defined as: ML (C) = reads (mC)/reads (mC + umC). This was corrected based on the bisulfite non-conversion rate reported previously (Lister et al., 2013). The corrected ML was then estimated as follows: ML_(correlated)_ = (ML – r)/(1 – r).

### Differentially Methylated Region Analysis

Differentially methylated regions (DMRs) were identified using DSS software (v.2.12.0) (Wu et al., 2015; Park and Wu, 2016) with the parameters including smoothing.span = 200, delta = 0, p.threshold = 1e-05, minlen = 50, minCG = 3, dis.merge = 100, pct.sig = 0.5. Based on the distribution of DMRs throughout the genome, genes were defined as DMR-related, if the genebody region (from the transcription start site, TSS, to the transcription end site, TES) or promoter region (2 kb upstream from the TSS) had overlaps with DMRs. GraphPad Prism software (ver. 8.0.1; GraphPad Software Inc., La Jolla, CA, United States) was used for the statistical analysis.

### Gene Ontology and Kyoto Encyclopedia of Genes and Genomes Enrichment Analysis of DMR-Related Genes

Gene Ontology (GO)^2^ enrichment analysis of genes related to DMRs was conducted using the GOseq R package to correct for gene length bias (Young et al., 2010). GO terms with corrected *P*-values < 0.05 were considered significantly enriched. Kyoto Encyclopedia of Genes and Genomes (KEGG)^3^ pathway enrichment analysis was conducted based on *P*-values < 0.05. KOBAS (v.3.0) software was used to test for enrichment of DMR-related genes in KEGG pathways (Mao et al., 2005; Kanehisa et al., 2008).

### Joint Analysis Between DNA Methylation and Gene Expression

Joint analysis was conducted between DNA methylation and gene expression, comparing resistant and susceptible poplar samples infected with the pathogen (6 dpi) to samples subjected to mock inoculation. The types of genes encoding methylation-related enzymes were analyzed based on previous research (Song et al., 2016). The DNA MLs of R, PR, and phytohormone genes were identified, with overlapping differentially expressed genes (DEGs) and DMRs considered to be associated with each other.

### Prediction of Potential Regulatory Pathway

miRNAs corresponding to target gene were predicted using psRNATarget^4^ and screened according to the following conditions: the gene appeared in combined analysis of DMRs and DEGs, and exhibited CHH hypomethylation in 5′ flanking region.

## Results

### Pathogen Infection and Methylation Profiles in *Populus tomentosa* and *Populus × euramericana*

*Lonsdalea populi* strain N-5-1, a Gram-negative, rod-shaped bacterium, causes a lethal disease known as bark canker in some poplars. Based on our previous reports, 6 dpi was selected as the time point for investigating lesions (Li et al., 2014; Hou et al., 2016). In this investigation, mock-inoculated and inoculated susceptible *P*. × *euramericana* and resistant *P*. *tomentosa* poplars were observed at 6 dpi. In *P*. × *euramericana*, the bark had a canker with white, odorous, and exudates dripping from the inoculation point and expanding lesions relative to mock-inoculation group (Supplementary Figures 1A,B,E,F). These symptoms were typical of *L*. *populi* infection (Li et al., 2014) but did not appear in the resistant *P*. *tomentosa* (Supplementary Figures 1C,D,G,H).

To investigate whether DNA methylation is involved in the poplar response to *L*. *populi* infection, whole-genome bisulfite sequencing (WGBS) of poplar bark from the mock-inoculation and inoculation groups was performed. In total, 19–26 million unique mapping clean reads on average were obtained for further analysis (Supplementary Table 1A). The bisulfite conversation rates of all samples were over 99% (Supplementary Table 1B). After methylation site calling, 3,911,428 and 3,702,079 mC on average were identified in *P*. *tomentosa* libraries by mock inoculation and at 6 dpi, accounting for 2.74 and 2.59% of all reads, respectively. In total, 7,896,041, 8,707,359, 6,821,148, and 6,332,367 mC on average were identified in *P*. × *euramericana* libraries by mock-inoculation, and at 2, 4, and 6 dpi, respectively, accounting for 4.43–6.09% of all reads (Supplementary Table 1C). To ensure the repeatability of the analysis, the correlations of MLs between samples were determined through Pearson correlation analysis. After inoculation, DNA methylation similarity to the control was higher for CG and CHG than CHH, in both poplar types. In addition, the *R*^2^ values of all samples were greater than 0.90 (Supplementary Figure 2).

### Dynamic Changes of DNA Methylation in Poplar During the Infection Process

**FIGURE 1:**
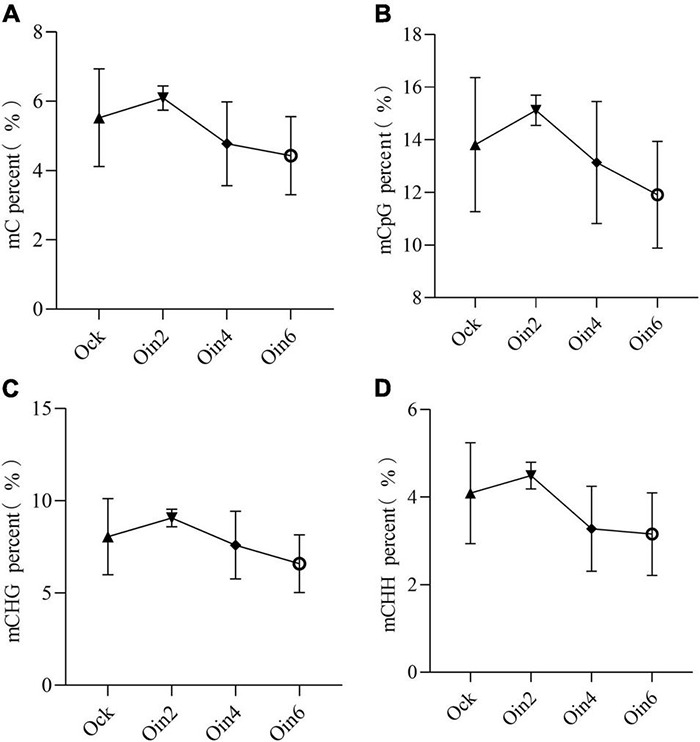
DNA methylation dynamic change tendency of *Populus* × *euramericana* “74/76” in response to *Lonsdalea populi* infection at different contexts. The mC percent **(A)**, mCpG percent **(B)**, mCHG percent **(C)**, and mCHH percent **(D)** of *P*. × *euramericana* in response to *L*. *populi* infection at different infection time points. The data were plotted as the mean ± SEM. Regular triangle, inverted triangle, diamond, and circle ring indicate the mean value of different infection time points. Oin/Ock means inoculation/mock-inoculation in *P*. × *euramericana*, and the Arabic numerals indicate the specific inoculation days.

Four successive disease development stages have been described for *L*. *populi* infection: the contact, penetration, incubation, and symptom appearance periods (Liu M. et al., 2015). The incubation period lasted approximately 3 days after the bacteria were inoculated onto the poplar stems (Shang et al., 2014). To explore the changes in DNA methylation among periods of *L*. *populi* infection, detection of DNA methylation variations in susceptible *P*. × *euramericana* was performed. After inoculation with *L*. *populi*, the methylation ratio of total cytosine was 6.09, 4.77, and 4.73% at 2, 4, and 6 dpi in *P*. × *euramericana*, respectively. Compared to the cytosine methylation ratio of the mock-inoculated group (5.53%), DNA methylation increased slightly at 2 dpi, while continuously decreased from 2 to 6 dpi. The result demonstrated that the proportion of total cytosine methylation differed among infection periods, indicating that gene expression in poplars was modulated in response to *L*. *populi* infection. This finding was consistent with previous reports that DNA methylation regulated plant gene expression in response to pathogen infection (Hewezi et al., 2017). The CG, CHG, and CHH methylation ratios increased slightly at 2 dpi and then decreased continuously, similar to the change trend of total cytosines and previous reports that DNA hypomethylation affected plant defense against nematodes (Figure 1; Atighi et al., 2020). In addition, the variation of CHH methylation was similar to that of total cytosines, with a sharp decline from 2 to 4 dpi and gradual decrease from 4 to 6 dpi. Meanwhile, the variation of CG and CHG methylation gradually decreased from 2 to 6 dpi. More importantly, compared to the mock-inoculation group, the decrease in methylation ratio was greatest for CHH. Overall, the level of DNA methylation initially increased, and then decreased, during the infection process in *P*. × *euramericana*.

**FIGURE 2:**
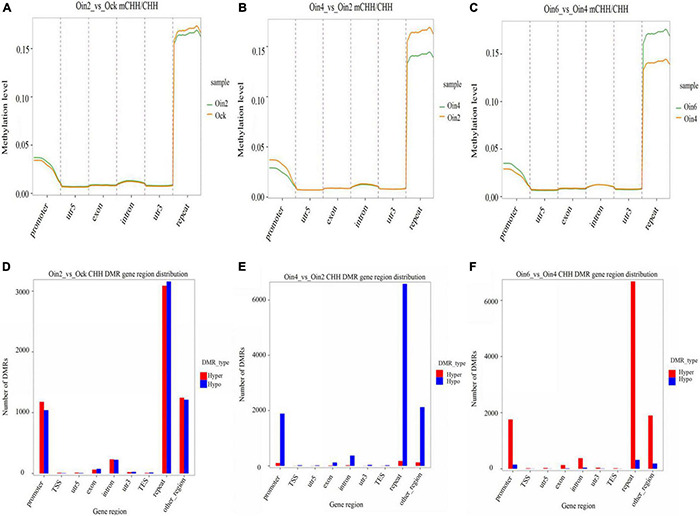
Changes in CHH methylation of *Populus* × *euramericana* ‘74/76’. Difference in CHH methylation level of *P*. × *euramericana* 2 dpi (Oin2) compared with mock-inoculation (Ock) **(A)**, 4 dpi (Oin4) compared with 2 dpi **(B)**, and 6 dpi (Oin6) compared with 4 dpi **(C)**; distribution of differentially methylated genes in *P*. × *euramericana* 2 dpi compared with mock-inoculation **(D)**, 4 dpi compared with 2 dpi **(E)**, and 6 dpi compared with 4 dpi **(F)**.

To elucidate how CHH methylation changed with the extension of infection course, we further analyzed CHH ML in genebody regions in *P*. × *euramericana*. Through pairwise analysis (Oin2_vs_Ock, Oin4_vs_Oin2 and Oin6_vs_Oin4), CHH methylation was compared among the different stages of disease development. DNA methylation region occurred mainly in the repeat region followed by the promoter region. Methylation was low in the intron, exon, 5′ untranslated region (UTR), and 3′ UTR (Figures 2A–C). This pattern was seen when comparing all infection conditions against the mock treatment (Figure 2A and Supplementary Figures 3A,B). Although the level of CHH methylation was lowest at 4 dpi, it still occurred mainly in the repeat and promoter regions (Figures 2B,C). Compared to mock inoculation, hyper and hypo CHH DMR genes had a roughly equal number at 2 dpi (Figure 2D). Interestingly, a large number of DMR genes exhibited CHH hypomethylation from 2 to 4 dpi (Figure 2E). In contrast, numerous DMR genes exhibited CHH hypermethylation from 4 to 6 dpi (Figure 2F). Relative to the control, the difference between hypo- and hypermethylated CHH DMR genes was most apparent at 4 dpi (Figures 2D, 5B, and Supplementary Figure 3C). Together, these results indicated that DNA methylation was lowest at 4 dpi, and that CHH DMRs were mainly in repeat and promoter regions in *P*. × *euramericana*. Additionally, we randomly chosen 200 random regions (RRs) from each of 19 chromosomes of poplar with each RR 150 bp in length to explore DMRs in RRs. The RRs were defined and chosen as candidates if they had overlapping hits with identified DMRs. Based on these 3,800 RRs results (Supplementary Table 2A), the number of DMRs for expectation in different categories of Figures 2D–F was further *in silico* predicted. It showed that the number of DMRs for observation differed from that for expectation identified based on the RRs in genome, especially DMRs in repeat regions were more, compared to the expectation values (Supplementary Table 2B).

Furthermore, CHH DMRs were annotated within the genebody and promoter regions to explore conserved and specific DMRs annotated genes of *P*. × *euramericana* under different conditions. A total of 70 conserved DMR-annotated genes were found in the genebody region during different infection periods, while 153 in gene promoter region (Oin2_vs_Ock, Oin4_vs_Ock, and Oin6_vs_Ock, Supplementary Figures 4A,C). Additionally, 334, 186, and 243 specific genes were found in the genebody region in pairwise comparisons (Oin2_vs_Ock, Oin4_vs_Oin2, and Oin6_vs_Oin4), respectively (Supplementary Figure 4B). There were 1,598, 981, and 1,059 specific genes in gene promoter region in Oin2_vs_Ock, Oin4_vs_Oin2, and Oin6_vs_Oin4 comparisons, respectively (Supplementary Figure 4D). Interestingly, conserved and specific DMR-annotated genes were more abundant in gene promoter region than that in genebody region, which was similar to our finding of more CHH DMRs in gene promoter region than that in genebody region.

### DNA Methylation in Two Poplar Types With Different Levels of Resistance to *Lonsdalea populi* Infection

**FIGURE 3:**
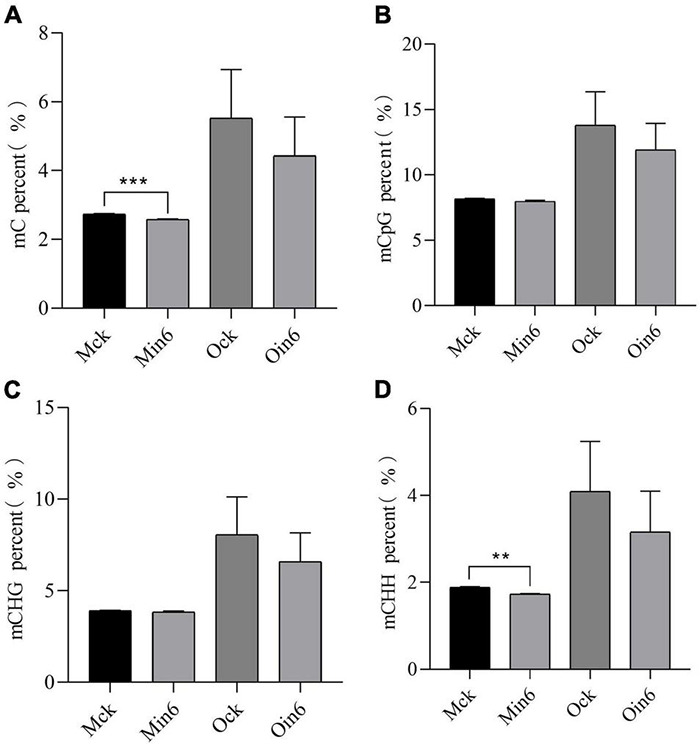
*Lonsdalea populi* infection induced methylation decline in different poplars. The mC percent **(A)**, mCpG percent **(B)**, mCHG percent **(C)**, and mCHH percent **(D)** of *P*. *tomentosa* and *P*. × *euramericana* before and after pathogen infection. The data are plotted as mean ± SEM. For statistical analysis of differentially expressed genes, unpaired *t*-test was performed. ***P < 0.001, **P < 0.01. Min/Oin means inoculation of *P*. *tomentosa* or *P*. × *euramericana*, Mck/Ock means mock-inoculation of *P*. *tomentosa* or *P*. × *euramericana*, and the Arabic numerals indicated the specific inoculation days.

To further explore the role of DNA methylation involved in the different resistances of poplars, susceptible *P*. × *euramericana* and resistant *P*. *tomentosa* were inoculated with *L*. *populi*. For mock-inoculation, the DNA ML of resistant type was much lower than that of susceptible type, suggesting the basal DNA methylation difference might be related to differences in the immune response. Interestingly, a sharp decrease in methylation occurred in *P*. *tomentosa* infected with *L*. *populi*, and significant decreases were observed in the percentages of both mC and mCHH (Figure 3), but not in mCHG. DNA methylation also decreased, although not significantly, for mCHH, mCHG, and mCG in *P*. × *euramericana* at 6 dpi (Figure 3). DNA methylation percentages were lower for the resistant than susceptible type in all three sequences, and with both mock inoculation and inoculation. The result showed that DNA methylation decreased significantly in *P*. *tomentosa* infected with *L*. *populi*, but not in *P*. × *euramericana*. CHH methylation plays an important role in disease resistance against pathogens (Geng et al., 2019). To further identify the methylation characteristics of various sequences in the genebody, the percent of mCpG, mCHG, and mCHH were analyzed. The decreasing trend of CHH methylation was consistent with the general trend of DNA hypomethylation (Figure 3), suggesting that CHH methylation was an important component of DNA methylation in the poplar response to pathogen infection, as observed in other plants.

### Global DNA Methylation Activated in Poplars in Response to *Lonsdalea populi* Infection

**FIGURE 4:**
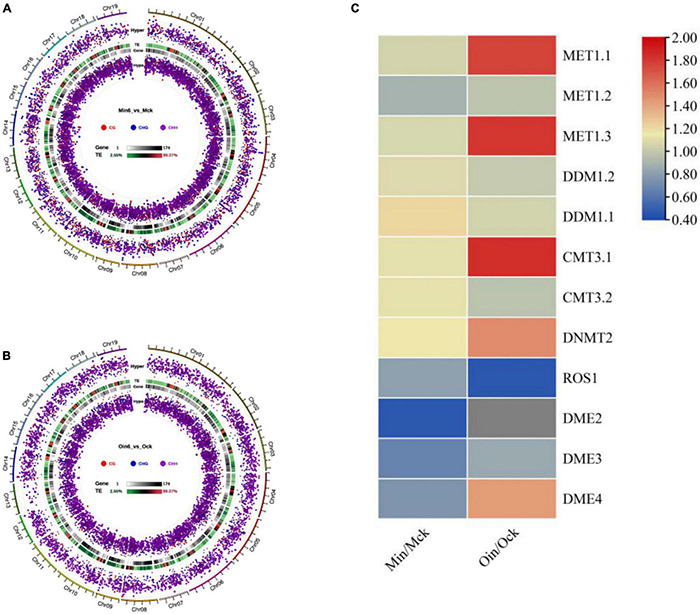
Distribution and significance of differential methylation regions (DMRs) in genome in three sequence contexts (CG/CHG/CHH) of two poplar types by Circos map. Display of three sequence contexts DMRs overall Circos of *P*. *tomentosa* (Min6_vs_Mck) **(A)**, *P*. × *euramericana* (Oin6_vs_Ock) **(B)**. Heatmap of hierarchical clustering for expression of DNA methylation- and demethylation-related genes in poplar under *Lonsdalea populi* infection **(C)**. In panels **(A,B)**, from the outside to the inside means: (a) Chromosomes. (b) Hyper differentially methylated regions (DMRs) statistical value log5 (| area stat|); the higher the outward dot, the more significant the position difference. (c) TE, the proportion of repeat elements. (d) Gene density. (e) Hypo DMR statistical value log5 (| area stat|); the higher the inward dot, the more significant the position difference.

To identify DMRs, we analyzed differential DNA hypomethylation and hypermethylation regions in CG, CHG, and CHH in two poplar types at 6 dpi. The numbers of differential hypomethylation regions of CG, CHG, and CHH in *P*. *tomentosa* were 1,183, 1,243, and 5,899, respectively, while the differential hypomethylation regions in *P*. × *euramericana* numbered 284, 653, and 5,594, respectively. Meanwhile, the numbers of differential hypermethylation regions of CG, CHG, and CHH in *P*. *tomentosa* were 921, 1,211 and 1,829, respectively, and the corresponding counts in *P*. × *euramericana* were 227, 460, and 3,700. Although CG and CHG hypomethylation occurred at nearly equal levels, regions of CHH hypomethylation were much more common in both poplars than regions of CHH hypermethylation. Interestingly, the number of differential CHH hypermethylation regions in *P*. × *euramericana* was approximately double that in *P*. *tomentosa* (Figures 4A,B). These data demonstrated that the infection suppressed the methylation of CHH and suggested that CHH hypomethylation could play a role in genetic regulation in plants, including poplar in response to pathogen infection.

To further examine the genetic changes that occurred in poplar during *L*. *populi* infection, the expression of genes encoding methylation-related enzymes was analyzed. In most plants, four classes of DNA methyltransferases participate in the establishment and maintenance of DNA methylation, and two classes of DNA glycosylases are responsible for demethylation. In this investigation, we analyzed the expression of 14 DNA methyltransferase- and glycosylase-related genes in resistant and susceptible poplars at 6 dpi under *L*. *populi* infection (Supplementary Table 3). The transcript levels of methylation-related genes increased, including *METHYLTRANSFERASE 1*.*1* (*MET1*.*1*) and *DECREASE IN DNA METHYLATION* (*DDM1*), excepting that expression of *MET1*.*2* slightly decreased. Demethylation-related genes including *REPRESSOR OF SILENCING 1* (*ROS1*) and *DEMETER* (*DME*) were repressed in both poplars, although the expression of *DME4* increased in *P*. × *euramericana*. Intriguingly, in *P*. *tomentosa*, genes related to the establishment and maintenance of DNA methylation were induced, including *CMT3*.*1*, *CMT3*.*2*, *MET1*.*1*, and *DNMT2*; the corresponding genes induced in *P*. × *euramericana* were *CMT3*.*1*, *MET1*.*1*, *DNMT2*, and *DME4*. Therefore, *L*. *populi* infection induced different patterns of methylation-related enzyme gene activation in *P*. × *euramericana* and *P*. *tomentosa* (Figure 4C). Differential expression of genes encoding methylation-related enzymes supported the occurrence of DNA methylation changes in poplar upon pathogen infection, which gave an impetus for poplar response to biotic stress.

### Gene Ontology Functional Classification and Kyoto Encyclopedia of Genes and Genomes Pathway Enrichment Analysis

In this investigation, GO annotation was used to classify the functions of CHH DMR genes. Based on WGBS data, genes showing DNA methylation were classified into three categories, “molecular function,” “biological process,” and “cellular component” (Supplementary Figure 5). In *P*. *tomentosa* (Min6_vs_Mck), 78 DMR genes were significantly enriched in the “biological process” category (Supplementary Table 4A). However, only 21 DMR genes in *P*. × *euramericana* (Oin6_vs_Ock) were significantly classified into “molecular function” group (Supplementary Table 4B), and no DMR genes were significantly enriched in “biological process.” These GO significant enrichment results indicate differing responses between resistant and susceptible poplar types.

To further explore the biological functions of poplar genes involved in the response to *L*. *populi* infection, the CHH DMRs were mapped to reference pathways in the KEGG database. In *P*. *tomentosa* (Min6_vs_Mck), 275 CHH DMRs were assigned to 79 pathways, including four genes significantly enriched in monoterpenoid biosynthesis pathway (Supplementary Table 5A). In *P*. × *euramericana* (Oin6_vs_Ock), 205 CHH DMRs were enriched in 71 pathways, including six genes significantly enriched in alpha-linolenic acid metabolism and 31 significantly enriched in biosynthesis of secondary metabolites (Supplementary Table 5B). Taken together, the significant enrichment of CHH DMR genes in monoterpenoid biosynthesis pathway supported the occurrence of systemic acquired resistance (SAR) in plants, suggesting that the resistant type might ward off pathogens via CHH DNA methylation modification of monoterpenoid biosynthesis genes (Riedlmeier et al., 2017).

### CHH Hypomethylation and Hypermethylation Are Concentrated in the Promoter and Repeat Regions of Both Poplar Types

**FIGURE 5:**
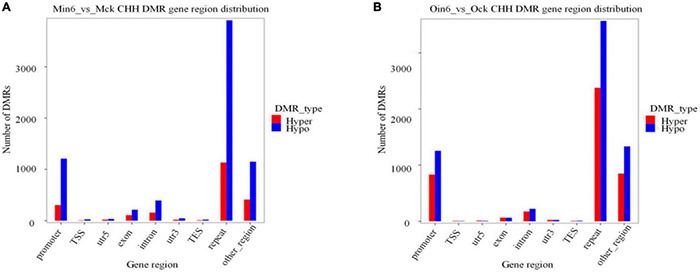
Number of CHH DMRs distributed in gene regions. Distribution of differentially methylated genes in *P*. *tomentosa* **(A)** and *P*. × *euramericana* **(B)** at 6 dpi compared with mock-inoculation. The abscissa represents the category of each area, and the ordinate represents the number of DMRs of Hyper/Hypo DMR in each region.

Cytosine methylation mostly resided in repetitive sequences in plants, mammals, and fungi (Goll and Bestor, 2005). To further analyze the methylation distribution among regions of gene bodies, we annotated DNA methylation differences in diverse parts of genes. Considering genotype-specific differences in methylation, CHH DMR gene region distribution of mock-inoculated samples was compared in two types of poplars (Ock vs. Mck) (Supplementary Figure 6). In the repeat region, 1,832 hypo DMRs and 1,959 hyper DMRs were found. The promoter region contained 1,372 hypo DMRs and 993 hyper DMRs. Together, these results showed no apparent difference in repeat or promoter region between resistant and susceptible poplars. We found that the most abundant CHH hypomethylation region in *P*. *tomentosa* (Min6 vs. Mck) was the repeat region (3,905 DMRs), followed by promoter region (1,208 DMRs). The same pattern was observed in *P*. × *euramericana* (Oin6 vs. Ock), with 3,567 hypo DMRs in repeat region and 1,252 in promoter region (Figure 5). In contrast, CHH methylation in exon, intron, or other regions in both poplars was much lower than that of repeat or promoter regions. These data demonstrated that a large number of promoter and repeat gene regions were CHH hypomethylated in both poplars, implying that CHH hypomethylation might be involved in poplar response to pathogen. Additionally, 1,133 DMRs were found in the repeat region, and 302 in the promoter region, in the analysis of CHH hypermethylation in *P*. *tomentosa*. Meanwhile, CHH hypermethylation in the *P*. × *euramericana* repeat and promoter regions included 2,376 and 830 DMRs, respectively. Nevertheless, the level of CHH hypermethylation in the promoter and repeat regions of *P*. × *euramericana* was approximately double that of *P*. *tomentosa*, further suggesting that CHH hypermethylation might be related to the difference in resistance between these poplars other than CHH hypomethylation.

### Correlation Analysis Between DNA Methylation and Gene Expression

DNA methylation plays an important role in regulating the expression of genes (Wang et al., 2015; Gao et al., 2020). To assess whether DNA methylation is associated with gene activity, the correlations between DNA methylation and gene expression were analyzed. The relationship between the ML of DMR regions and the levels of associated DEGs was visualized as a scatter diagram (Supplementary Figures 7A,B). Intergroup comparison showed more genes of *P*. × *euramericana* in each quadrant than that of *P*. *tomentosa*. Furthermore, most of the interconnected genes showed hypomethylation compared to the control, in both poplars. To explore the CHH methylation distribution in more depth, we analyzed promoter DNA hypomethylation. The relationship between methylation and DMR-related gene expression was illustrated using scatter and box plots (Supplementary Figures 7C,D). In resistant type poplar, CHH hypomethylation level in the promoter region was negatively correlated with the expression level of DMR-related genes (Min6 vs. Mck). This correlation was relatively weak in the susceptible poplar.

**FIGURE 6:**
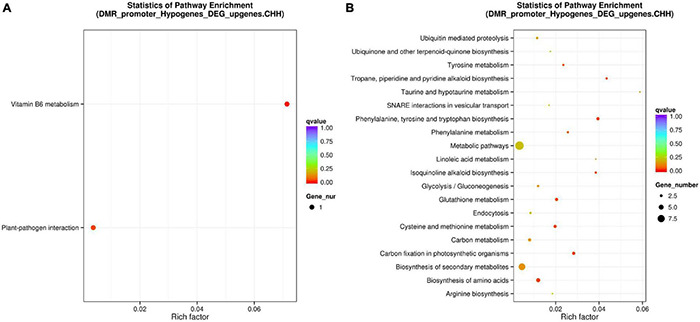
Combined analysis of promoter region CHH hypomethylation and differential gene expression of *Populus tomentosa* ‘henan’ and *Populus* × *euramericana* ‘74/76’. Scatter plot of enriched KEGG metabolic pathway of the intersection of DNA CHH hypo-DMRs for Min6 vs. Mck **(A)** and Oin6 vs. Ock **(B)** in promoter region and up regulated DEGs. For panels **(A,B)**, the vertical axis represented the pathway name, the horizontal axis represented the richness factor, the size of the dot represented the number of DMR-related genes in this pathway, and the color of the dot corresponded to different Q value ranges.

Although promoter DNA methylation promoted gene transcription in some cases, it usually negatively regulated gene transcription (Zemach et al., 2010; Wang et al., 2015; Zhang et al., 2018). Promoter methylation has a negative correlation with gene expression levels (Xin et al., 2021). Therefore, we conducted combined analysis of DNA CHH hypo-DMRs in the promoter region (Min6 vs. Mck) and upregulated DEG transcriptome profiles in poplars generated under the same inoculation conditions. Five genes overlapping between the presence of DMRs and DEGs were found in *P*. *tomentosa* (Table 1). Through analysis of those five genes using the KEGG database, we found that two genes were enriched in the plant–pathogen interaction pathway (Supplementary Figure 8) and Vitamin B6 (VitB6) metabolism pathway (Supplementary Figure 9), respectively (Figure 6A). Functional annotations of Potri.007G127000 and Potri.001G182100 were obtained from Phytozome, indicating that these genes encode CALCIUM-DEPENDENT PROTEIN KINASE 24 (*CDPK24*) and pyridoxin biosynthesis PDX1-like protein 2, respectively. The correlation between DMRs and DEGs indicated that *CDPK24*, a protein kinase gene regulated by calcium ions in the plant pathogen interaction pathway, and *PDX1*.*2*, a VitB6 metabolism related gene, might enhance plant disease resistance.

**TABLE 1:** Transcriptional up-regulated expression genes with downregulated CHH methylation in the promoter region of *Populus tomentosa* ‘henan’.

Gene ID	Gene name	Differential methylation	log_2_Fold Change	*P* value	Swiss prot annotation
Potri.001G182100	PDX12	–0.16	1.27	0.00	Pyridoxal biosynthesis protein PDX1.2
Potri.001G324500	CPD	–0.19	1.09	0.00	Cyclic phosphodiesterase
Potri.005G146400	NPY2	–0.17	1.33	0.00	BTB/POZ domain-containing protein NPY2
Potri.007G127000	CDPKO	–0.07	1.53	0.00	Calcium-dependent protein kinase 24
Potri.012G110600		–0.14	1.51	0.00	

In *P*. × *euramericana* (Oin6 vs. Ock), we found that 93 genes with DNA CHH hypo-DMRs in the promoter region were upregulated. Through comparison of these 93 genes against the KEGG enrichment database (Supplementary Table 6), we found that the two most abundant pathways were biosynthesis of secondary metabolites and metabolic pathways (Figure 6B). These genes and pathways in *P*. × *euramericana* differed markedly from those identified in *P*. *tomentosa*, which might be due to the abnormal metabolism of *P*. × *euramericana* under the influence of pathogen infection at 6 dpi (Figure 6).

### DNA Methylation Changes of Resistance, Pathogenesis-Related, and Phytohormone Genes

**FIGURE 7:**
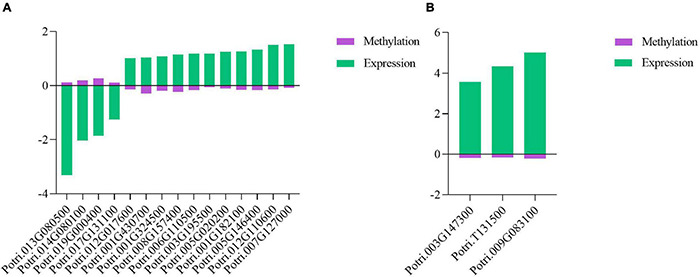
Co-relation between differentially expressed genes and differentially methylated regions in *R/PR* genes and phytohormone genes in poplar under pathogen infection. Co-relation between phytohormone DEGs and DMRs in *P*. *tomentosa* **(A)**. Co-relation between *R*/*PR* DEGs and DMRs in *P*. × *euramericana* **(B)**.

Phytohormone genes, *R* genes, and *PR* genes are critical to plant defenses against pathogen infections (Derksen et al., 2013; Verma et al., 2016; Wang et al., 2018; Zou et al., 2018; Yang et al., 2021). To explore the DNA methylation characteristics of these genes, combined analysis of DMRs and DEGs was performed. In this analysis, 15 phytohormone-related DEGs, including one *R* gene in *P*. *tomentosa*, were found to be associated with differential DNA methylation, and the majority of these DEGs exhibited DNA hypomethylation, suggesting that they contributed to poplar defense against the pathogen infection (Figure 7A). In *P*. × *euramericana*, 214 phytohormone-related DEGs (Supplementary Table 7) including one *R* and two *PR* genes (Figure 7B) were involved in poplar response to biotic stress. Of these DEGs, 121 exhibited DNA hypomethylation and upregulated expression, while the others showed DNA hypermethylation and downregulated expression. These results implied that differentially expressed *R*, *PR*, and phytohormone genes might be modified by DNA methylation.

### miRNA Methylation in Poplars With Different Resistance Levels

**FIGURE 8:**
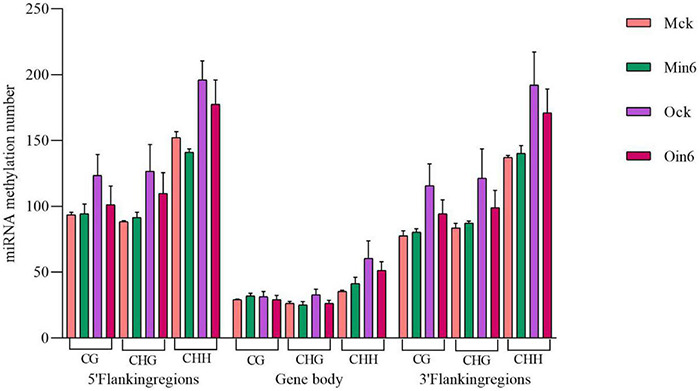
miRNA methylation numbers under different contexts in different regions of *Populus tomentosa* ‘henan’ and *Populus × euramericana* ‘74/76’. Error bars indicate SEM.

Considering the important roles of miRNAs in biotic stress responses, the 5′ flanking region, genebody, and 3′ flanking region of miRNA were analyzed to identify whether there were regulation of DNA methylation on miRNAs in poplar. We analyzed miRNA methylation in different regions under CG, CHG, and CHH contexts in both *P*. *tomentosa* (Mck, Min6) and *P*. × *euramericana* (Ock, Oin6). Intriguingly, the results showed that the pathogen infection induced miRNA hypomethylation of all regions in *P*. × *euramericana*. However, apparent miRNA CHH hypomethylation was detected only in the 5′ flanking regions in *P*. *tomentosa*, and no changes were apparent in other regions under different contexts (Figure 8). Overall, pathogen infection reduced the number of CHH-methylated miRNAs in the 5′ flanking region of *P*. *tomentosa*, and the number of methylated miRNAs in *P*. × *euramericana*, indicating that miRNA methylation might be involved in regulating the response to *L*. *populi* infection in poplars.

## Discussion

### DNA Methylation Is Involved in the Defense of Poplar to Pathogen Infection

Plants undergo genome-wide DNA methylation changes during infection with pathogens, which has been described in a few annual plants (Zhang et al., 2018). For example, global DNA hypomethylation was suggested to be part of the basal pattern-triggered immunity response in rice and tomato treated with different nematode species or flag22 (Atighi et al., 2020). Similarly, DNA hypomethylation was associated with resistance of *P*. *tomentosa* to *L*. *populi*, suggesting that pathogen-induced DNA methylation may involve similar mechanisms between annual and perennial plants. In response to *Heterodera glycines* infection, the susceptible soybean line exhibited reduced global methylation of both protein-coding genes and transposable elements, whereas the resistant line showed the opposite response, with increased global MLs (Rambani et al., 2020). When a resistant near-isogenic line of wheat was compared to a susceptible line, more hypermethylated and fewer hypomethylated genes were found in the former line at both 0 and at 96 h post-inoculation with *Puccinia triticina* (Saripalli et al., 2020). Those results suggest that regulation of DNA methylation is of great significance for plants facing biotic stress. In this investigation, DNA methylation changes associated with pathogen infection between two poplar types with different resistance levels were investigated through WGBS. Interestingly, both resistant and susceptible poplars showed DNA hypomethylation; the previously reported contrasting DNA methylation patterns were not observed here (Figure 3; Rambani et al., 2020; Saripalli et al., 2020). Intriguingly, the resistant type *P*. *tomentosa* showed significantly reduced DNA hypomethylation under pathogen stress, which differed from the DNA hypermethylation patterns observed in resistant plants in previous studies. These results suggested that the DNA methylation patterns of susceptible and resistant types may differ, making it necessary to explore the subtle differences in epigenetic regulation between annual and perennial plants during pathogen infection.

Cyst nematode parasitism induced dynamic changes in the *Arabidopsis* root epigenome, evidenced by a clear distinction between two infection time points, with increased CG hypermethylation seen at 10 dpi relative to 5 dpi, specifically in protein-coding genes (Hewezi et al., 2017). Similarly, this investigation indicated dynamic changes in DNA methylation of poplars, which were associated with different courses of pathogen infection (Figure 1). Interestingly, our results differed from the DNA methylation changes observed in *Arabidopsis*, which were largely similar between 3 and 5 dpi after infection with *Pseudomonas syringae* (Dowen et al., 2012). DNA methylation decreased with prolongation of infection time in poplar (Figure 1), in contrast to the trend reported in the *Arabidopsis* root epigenome (Hewezi et al., 2017). The change trend of DNA methylation in this investigation could be elucidated more thoroughly using four rather than two infection time points (Dowen et al., 2012; Hewezi et al., 2017). Comprehensively, there might be a specific relationship between the pathogen infection period and global changes in DNA methylation may differ among plant species and stressors.

The methylation maintenance mode and structure of CHH differed from the other two sequence contexts, indicating that the regulation process of CHH methylation in plants might be different with that of CG and CHG. Our results provided compelling evidence that CHH methylation might be the main type of DNA methylation occurring in poplars under biotic stress, and it changed dynamically among infection time points (Figure 1). This was consistent with previous reports that CHH hypomethylation played key roles in plant immune responses (Geng et al., 2019; Sun et al., 2019; Atighi et al., 2020). In this investigation, the number of differential CHH hypermethylation sited in *P*. × *euramericana* was roughly double that in *P*. *tomentosa* (Figure 5). Moreover, a more significant difference in CHH methylation occurred in *P*. *tomentosa* (Figure 3). Together, these results reflected dynamic variation of CHH hypomethylation between different poplar species, demonstrating the key role of CHH hypomethylation in the poplar immune response to pathogen affection. Further annotation of DMR regions revealed that the level of CHH hypomethylation in susceptible and resistant poplars was overwhelmingly higher in repeat regions, followed by promoter regions, which was different with the previous finding that CHH hypomethylation occurred predominantly in gene promoters during the rice immune response to *Meloidogyne graminicola* infection (Atighi et al., 2020). Similarly, in wheat inoculated with *P*. *triticina*, ML was generally abundant in intergenic regions, followed by promoter regions, transcription termination sites, and exons/introns (Saripalli et al., 2020). Overall, these investigations supported the conclusion that the promoter is a crucial region for DNA methylation mediated plant responses to biotic stressors, even though the promoter region may not accumulate the highest level of DNA methylation.

### Methylation of Genes Encoding CDPK and PDX Is Involved in the Regulation of the Poplar Defense Response

Previous reports have suggested that many *CDPKs* were associated with plant defense mechanisms against abiotic attacks. *CDPK* genes and the CDPK-related protein kinases (CRKs) played pivotal roles in the biological processes underlying *Arabidopsis* immunity to bacteria, fungi, insects, and viruses (Yip Delormel and Boudsocq, 2019). *CDPK* and *CRK* genes, which were dramatically induced during *Ralstonia solanacearum* infection, may act in a coordinated manner to mediate the immune response of pepper plants (Cai et al., 2015). Many rice *CDPK* genes had been demonstrated to respond to various stresses, including rice blast and chitin stress (Wan et al., 2007). Furthermore, a few *CDPK* genes were differentially expressed in *P. trichocarpa* during fungal infection, according to genome-wide analysis of the *CDPK* gene family (Zuo et al., 2013). The present investigation suggested a crucial role of *CDPK24* in the defense response of poplars to pathogen infection, based on analysis of whole-genome DNA methylation and transcriptomic profiles. We found that CHH hypomethylation stimulated the expression of *CDPK24*, which was involved in the immune response of the resistant poplar to pathogen infection, thereby providing the first insight into the crucial role of DNA methylation in modifying *CDPK24* in the poplar response to biotic stress. Considering that CDPKs were related to immune responses in diverse plants, the regulatory roles of CDPKs in plant responses to pathogen infection may be conserved. However, the detailed functions of CDPKs in pathogen resistance remain to be confirmed.

Vitamins are essential nutrients and key enzyme cofactors that regulate cellular metabolism and activate the immune system. Recently, B vitamins have been shown to play roles in the development, stress tolerance, and pathogen resistance of plants (Suzuki et al., 2020). Other studies have obtained evidence through expression profiling of genes involved in VitB6 biosynthesis, showing their involvement in plant disease resistance. *Bacillus subtilis* CBR05 was reported to induce VitB6 biosynthesis in tomato plants through a *de novo* pathway, contributing to resistance against *Xanthomonas campestris* infection (Chandrasekaran et al., 2019). Moreover, Vitamin B6 contributed to disease resistance against *Pseudomonas syringae* and *Botrytis cinerea* in *Arabidopsis* (Zhang et al., 2015; Chandrasekaran et al., 2019). Transcriptome sequencing indicated that the VitB6 biosynthesis pathway was involved in the response of *Lilium pumilum* to *Fusarium oxysporum* (He et al., 2019). Two protein families, PDX1s and PDX2, were required for the *de novo* biosynthesis of VitB6 (Tambasco-Studart et al., 2007). In this investigation, the VitB6 metabolism pathway was found to contribute to the resistance of poplars to pathogen infection. Specifically, this investigation unraveled that DNA methylation modification of *PDX* was involved in the poplar response to pathogen infection, providing new insights into the connection between the VitB6 metabolism pathway and pathogen resistance in poplars.

Furthermore, two miRNAs targeting *CDPK24* were further predicted, namely, ptr-miR477d and ptr-miR169n (Supplementary Table 8). MiR477d-5p was downregulated in *P*. *tomentosa* upon pathogen infection, which was consistent with expectation that it showed CHH hypomethylation in the 5’ flanking regions. Previous reports revealed that miR477 was related to plant resistance to pathogen (Hu et al., 2020; Wang et al., 2020). Thus, infection with *L*. *populi* in *P*. × *euramericana* and *P*. *tomentosa* should trigger a battery of plant immune responses. We further postulated that CHH hypomethylation might trigger the suppression of ptc-miR477d-5p, thereby stimulating the induction of *CDPK24* and indicating that ptc-miR477d-5p and its target, *CDPK24*, enhanced the plant immune response to pathogen infection in poplars. Additionally, *PDX1*.*2* was involved in the defense of poplar plants to against biotic stress (Supplementary Figure 10).

### DNA Methylation Affects Poplar Responses to Biotic Stress Through Multiple Pathways

Phytohormones had been confirmed to function as regulators of plant immune responses to biotic stress through exogenous hormone treatment (Robert-Seilaniantz et al., 2011). *R* and *PR* genes were responsible for plant resistance to multiple diseases (Tyr and Rebecca, 2016). In this investigation, nearly half of the plant hormone genes were associated with DMRs and DEGs, and a negative correlation was found between DNA hypomethylation and differential gene expression (Figure 7 and Supplementary Table 7). In addition, all *R* and *PR* genes associated with DEGs and DMRs exhibited DNA hypomethylation and upregulated expression. These *R* and *PR* genes and phytohormone related DEGs participated in the immune response through DNA methylation modification, suggesting that DNA methylation modulated poplar defense against pathogen infection through modification of *R*, *PR*, and phytohormone genes. In grape berries, melatonin treatment enhanced disease resistance and flavonoid biosynthesis by decreasing the MLs of the promoters of the corresponding genes (Gao et al., 2020). Heterologous expression of the lycopene β-cyclase (*lcb*) gene in flax was reported to silence its endogenous counterpart due to changes in gene-body methylation and the abscisic acid homeostasis mechanism, thereby increasing plant resistance to fungal pathogens (Boba et al., 2018). Combined together, these results indicated that DNA methylation effectively functioned in plant disease defense through modification of *R*, *PR*, and phytohormone genes, thus helping to modulate the molecular epigenetic mechanism.

**FIGURE 9:**
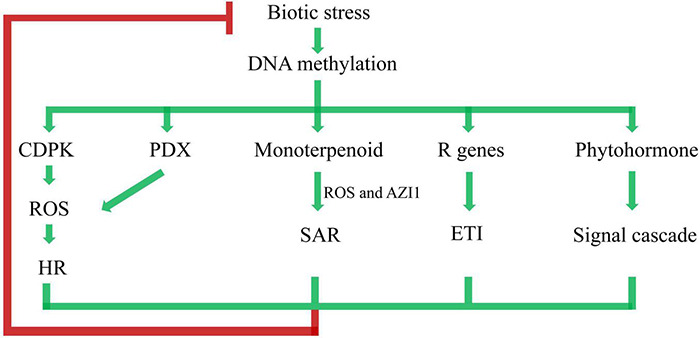
Network of immune pathways associated with plant–pathogen interaction. ROS represents reactive oxygen species, HR represents hypersensitive response, AZI1 represents *AZELAIC ACID INDUCED1*, SAR represents systemic acquired resistance, ETI represents effector-triggered immunity. Arrows represent positive regulation (accumulation of transcripts, proteins, or hormones), and blocked arrow represents negative regulation.

To systematically understand the crosstalk of DNA methylation and poplar response to biotic stress, we further postulated a putative regulation model (Figure 9). Biotic stress triggered DNA methylation changes, which was followed by a wide range of response activities. The plant-pathogen interaction pathway was activated by CDPK. Similarly, the VitB6 metabolism pathway was activated by PDX. CDPK and PDX may regulate a burst of reactive oxygen species (ROS), leading to the hypersensitive response (HR) (Tambasco-Studart et al., 2007). Appropriate activation of HR by pathogens may cause, or have an association with, plant disease resistance (Balint-Kurti, 2019). Enrichment of the monoterpenoid biosynthesis pathway in the KEGG results indicated SAR through ROS and *AZELAIC ACID INDUCED1* (AZI1), which likely functioned as infochemicals in plant-to-plant signaling, thereby allowing defense signals to propagate between neighboring plants (Riedlmeier et al., 2017). Effector-triggered immunity (ETI) is triggered by the activation of *R* genes, resulting in halting further colonization and attenuating disease resistance (Jones and Dangl, 2006). Phytohormone-regulating signal cascades were involved in poplar responses to biotic stresses (Bari and Jones, 2009; Verma et al., 2016). Taken together, as perennial and sessile organisms, poplars are equipped with a sophisticated multilayered immune system based on DNA methylation to win the arm race with pathogen infection.

## Data Availability Statement

The datasets presented in this study can be found in online repositories. The names of the repository/repositories and accession number(s) can be found below: NCBI SRA BioProject, accession numbers: PRJNA778554, PRJNA778562, PRJNA778625, PRJNA778867, PRJNA779116, and PRJNA779165.

## Author Contributions

DX was responsible for conceptualization, bioinformatic analysis, data interpretation, and drafting of the manuscript. KZ performed the pathogen culture and inoculation and was responsible for sequencing. XY and YM assisted with the data analysis and critical evaluation of the manuscript. YY contributed to the plant growth, sampling, and DNA extraction. YW was responsible for supervision, project administration, and funding acquisition to support this research. All authors have read and agreed to submit the manuscript.

## Conflict of Interest

The authors declare that the research was conducted in the absence of any commercial or financial relationships that could be construed as a potential conflict of interest.

## Publisher’s Note

All claims expressed in this article are solely those of the authors and do not necessarily represent those of their affiliated organizations, or those of the publisher, the editors and the reviewers. Any product that may be evaluated in this article, or claim that may be made by its manufacturer, is not guaranteed or endorsed by the publisher.

## Footnotes

1
https://phytozome.jgi.doe.gov/pz/portal.html#!info?alias=Org_Ptrichocarpa

2
http://www.Geneontology.org/

3
http://www.genome.jp/kegg/

4
http://plantgrn.noble.org/psRNATarget/
