# Supplementary material for: Crosstalk of DNA Methylation Triggered by Pathogen in Poplars With Different Resistances
Source: Front Microbiol. 2021 Dec 28;12:750089. doi: 10.3389/fmicb.2021.750089 (PMC8748266; doi:10.3389/fmicb.2021.750089)
Supplement: Supplementary file 1 [file Table_1.DOCX]

**Supplementary Table 1** DNA methylation sequencing data.

A Summary of bisulphate sequencing samples and results

| **Samples** | **Total reads** | **Mapped reads** | **Mapping rate**  **(%)** | **Duplication rate**  **(%)** |
| --- | --- | --- | --- | --- |
| Mck_a | 65580851 | 20539922 | 31.32 | 12.85 |
| Mck_b | 65453743 | 20369204 | 31.12 | 13.9 |
| Mck_c | 65282438 | 20818569 | 31.89 | 12.18 |
| Min6_a | 65570452 | 20379296 | 31.08 | 12.71 |
| Min6_b | 65644343 | 19791769 | 30.15 | 12.5 |
| Min6_c | 63779597 | 19554824 | 30.66 | 12.52 |
| Ock_a | 62430309 | 18011144 | 28.85 | 12.46 |
| Ock_b | 72917776 | 32769248 | 44.94 | 6.34 |
| Ock_c | 65466969 | 28504318 | 43.54 | 12.3 |
| Oin2_a | 65639121 | 29117514 | 44.36 | 13.05 |
| Oin2_b | 57264042 | 24205510 | 42.27 | 12.24 |
| Oin2_c | 61288651 | 27144743 | 44.29 | 12.9 |
| Oin4_a | 65501522 | 17659210 | 26.96 | 13.27 |
| Oin4_b | 72376525 | 29167739 | 40.3 | 6.25 |
| Oin4_c | 63986341 | 24730720 | 38.65 | 6.15 |
| Oin6_a | 65267847 | 27190585 | 41.66 | 12.87 |
| Oin6_b | 65358702 | 21810198 | 33.37 | 11.91 |
| Oin6_c | 65331265 | 15561907 | 23.82 | 13.51 |

B DNA Quality of original data from methylation sequencing

| **Sample name** | **Raw reads** | **Raw bases (G)** | **Clean reads** | **Clean bases (G)** | **Clean ratio (%)** | **BS conversion**  **rate (%)** |
| --- | --- | --- | --- | --- | --- | --- |
| MCK_a | 66666666 | 20.00 | 65580851 | 17.97 | 89.85 | 99.55 |
| MCK_b | 66666666 | 20.00 | 65453743 | 17.87 | 89.35 | 99.61 |
| MCK_c | 66666666 | 20.00 | 65282438 | 17.83 | 89.15 | 99.55 |
| MIN6_a | 66666666 | 20.00 | 65570452 | 17.97 | 89.85 | 99.49 |
| MIN6_b | 66666666 | 20.00 | 65644343 | 18.02 | 90.1 | 99.58 |
| MIN6_c | 64901549 | 19.47 | 63779597 | 17.45 | 89.63 | 99.57 |
| OCK_a | 63825991 | 19.15 | 62430309 | 17.1 | 89.3 | 99.47 |
| OCK_b | 74302071 | 22.29 | 72917776 | 19.97 | 89.59 | 99.55 |
| OCK_c | 66666666 | 20.00 | 65466969 | 17.91 | 89.55 | 99.60 |
| OIN2_a | 66666666 | 20.00 | 65639121 | 18.03 | 90.15 | 99.54 |
| OIN2_b | 58406072 | 17.52 | 57264042 | 15.69 | 89.55 | 99.46 |
| OIN2_c | 62439826 | 18.73 | 61288651 | 16.81 | 89.75 | 99.48 |
| OIN4_a | 66666666 | 20.00 | 65501522 | 17.98 | 89.9 | 99.45 |
| OIN4_b | 73780483 | 22.13 | 72376525 | 19.84 | 89.65 | 99.48 |
| OIN4_c | 65170014 | 19.55 | 63986341 | 17.56 | 89.82 | 99.51 |
| OIN6_a | 66666666 | 20.00 | 65267847 | 17.85 | 89.25 | 99.53 |
| OIN6_b | 66666666 | 20.00 | 65358702 | 17.91 | 89.55 | 99.52 |
| OIN6_c | 66666666 | 20.00 | 65331265 | 17.89 | 89.45 | 99.59 |

C Summary of the methylation ratio of different samples

|  | **Methylated C** | **Unmethylated C** | **Ratio** |
| --- | --- | --- | --- |
| Mck_a | 3892189 | 138835470 | 2.72% |
| Mck_b | 3944224 | 138783435 | 2.76% |
| Mck_c | 3897872 | 138829787 | 2.73% |
| Min6_a | 3738881 | 138988778 | 2.61% |
| Min6_b | 3674334 | 139053325 | 2.57% |
| Min6_c | 3693021 | 139034638 | 2.58% |
| Ock_a | 3993872 | 138733787 | 2.79% |
| Ock_b | 10667799 | 132059860 | 7.47% |
| Ock_c | 9026452 | 133701207 | 6.32% |
| Oin2_a | 9505106 | 133222553 | 6.65% |
| Oin2_b | 7772929 | 134954730 | 5.44% |
| Oin2_c | 8844042 | 133883617 | 6.19% |
| Oin4_a | 3512136 | 139215523 | 2.46% |
| Oin4_b | 9344007 | 133383652 | 6.54% |
| Oin4_c | 7607300 | 135120359 | 5.32% |
| Oin6_a | 8761615 | 133966044 | 6.13% |
| Oin6_b | 6938521 | 135789138 | 4.86% |
| Oin6_c | 3296964 | 139430695 | 2.30% |

Note: M stands for *Populus tomentosa* `henan', O stands for *Populus* × *euramericana* `74/76', ck stands for mock-inoculation, in stands for inoculation, the arabic numerals indicate the specific inoculation days. a, b, c represent three different biological replicates.
