# Supplementary material for: Crosstalk of DNA Methylation Triggered by Pathogen in Poplars With Different Resistances
Source: Front Microbiol. 2021 Dec 28;12:750089. doi: 10.3389/fmicb.2021.750089 (PMC8748266; doi:10.3389/fmicb.2021.750089)
Supplement: Supplementary file 2 [file Table_2.DOCX]

**Supplementary Table 2** DNA methylation comparison analysis performed based on random DMRs.

**A** DMRs number in random regions of 3800 random regions (RRs).

|  | **Hyper** | **Hypo** |
| --- | --- | --- |
| Oin2_vs_Ock | 7 | 4 |
| Oin4_vs_Oin2 | 3 | 28 |
| Oin6_vs_Oin4 | 18 | 2 |

**B** Observation and expectation DMRs number in different categories.

|  | **Oin2_vs_Ock** | | **Oin4_vs_Oin2** | | **Oin6_vs_Oin4** | |
| --- | --- | --- | --- | --- | --- | --- |
|  | **Hyper** | **Hypo** | **Hyper** | **Hypo** | **Hyper** | **Hypo** |
| Promoter | 1178(1396) | 1041(931) | 102(698) | 1889(6514) | 1755(4187) | 144(465) |
| Exon | 61(1141) | 75(761) | 14(571) | 120(5327) | 132(3424) | 13(380) |
| Intron | 232(1409) | 223(940) | 21(705) | 370(6577) | 375(4228) | 38(470) |
| UTR5 | 12(123) | 6(82) | 3(61) | 15(573) | 24(369) | 3(41) |
| UTR3 | 21(232) | 24(155) | 2(116) | 34(1084) | 33(697) | 4(77) |
| Repeat | 3089(1036) | 3158(690) | 173(518) | 6583(4833) | 6679(3107) | 314(345) |

Note: Expected value of DMRs in each category was calculated as:

Expectation = DMRs number of RRs *$\frac{\text{total genomic chromosome length}}{\text{total RRs length}}$*$\frac{\text{category length}}{\text{total genomic chromosome length}}$

Category proportion of promoter, exon, intron, UTR5, UTR3, and repeat in the genome was 33.61%, 27.49%, 33.94%, 2.96%, 5.59%, and 24.94%, respectively.

Observation data in Supplementary Table 2 B corresponded to the Figure 2 panel D, E and F respectively. The values in brackets were the expected DMRs.
