# Supplementary material for: Crosstalk of DNA Methylation Triggered by Pathogen in Poplars With Different Resistances
Source: Front Microbiol. 2021 Dec 28;12:750089. doi: 10.3389/fmicb.2021.750089 (PMC8748266; doi:10.3389/fmicb.2021.750089)
Supplement: Supplementary file 8 [file Table_8.DOCX]

**Supplementary Table 8** The predict miRNAs target *CDPK24* using psRNATarget.

| **miRNA_Acc.** | **Target_Acc.** | **Expectation** | **UPE$** | **miRNA_start** | **miRNA_end** | **Target_start** | **Target_end** | **miRNA_aligned_fragment** | **alignment** | **Target_aligned_fragment** | **Inhibition** | **Target_Desc.** | **Multiplicity** |
| --- | --- | --- | --- | --- | --- | --- | --- | --- | --- | --- | --- | --- | --- |
| ptc-miR477a-5p | Your_Sequence | 4.0 | -1.0 | 1 | 21 | 415 | 435 | AUCUCCCUCAGAGGCUUCCAA | ::::: ..: ::::::::.: | AUGGAACUUUGUGAGGGAGGU | Translation |  | 1 |
| ptc-miR477b | Your_Sequence | 4.0 | -1.0 | 1 | 21 | 415 | 435 | AUCUCCCUCAGAGGCUUCCAA | ::::: ..: ::::::::.: | AUGGAACUUUGUGAGGGAGGU | Translation |  | 1 |
| ptc-miR477d-5p | Your_Sequence | 4.0 | -1.0 | 1 | 22 | 414 | 435 | AUCUCCCUCAAAGGCUUCCUCU | :::: ..: ::::::::.: | UAUGGAACUUUGUGAGGGAGGU | Translation |  | 1 |
| ptc-miR169n-5p | Your_Sequence | 4.5 | -1.0 | 1 | 21 | 839 | 859 | UGAGCCAAGGAUGACUUGCCG | ::::: ::::::::: .: | UCGCAAGGGAUCCUUGGCCUA | Cleavage |  | 1 |
| ptc-miR828a | Your_Sequence | 4.5 | -1.0 | 1 | 22 | 176 | 197 | UCUUGCUCAAAUGAGUAUUCCA | ::::: ::::::. :::: | AAAAAUACACAUUUGGAAAAGA | Cleavage |  | 1 |
| ptc-miR828b-5p | Your_Sequence | 4.5 | -1.0 | 1 | 22 | 176 | 197 | UCUUGCUCAAAUGAGUAUUCCA | ::::: ::::::. :::: | AAAAAUACACAUUUGGAAAAGA | Cleavage |  |  |
