# Supplementary figures and images for: Crosstalk of DNA Methylation Triggered by Pathogen in Poplars With Different Resistances
Source: Front Microbiol. 2021 Dec 28;12:750089. doi: 10.3389/fmicb.2021.750089 (PMC8748266; doi:10.3389/fmicb.2021.750089)

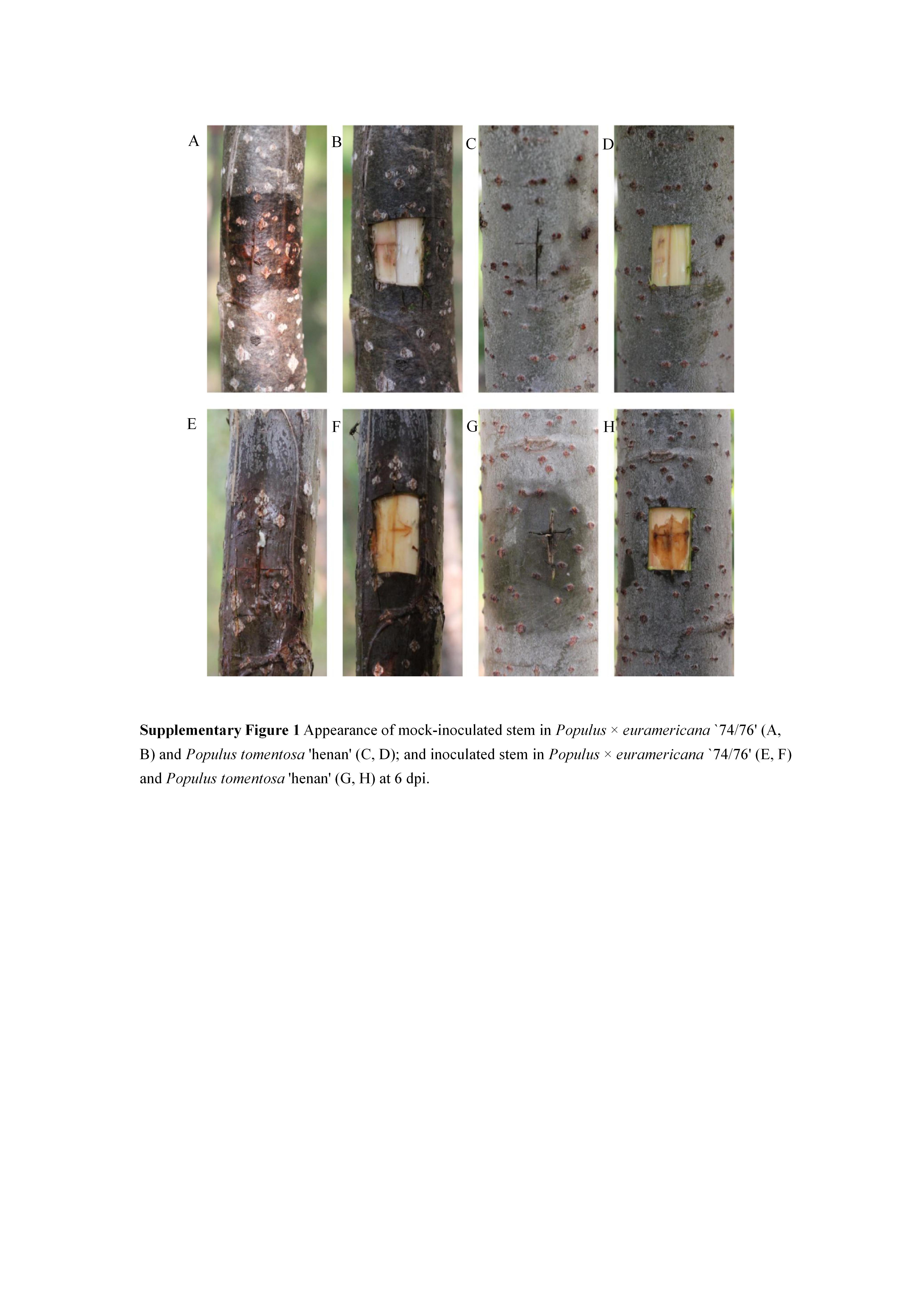

Supplement: Supplementary file 9 [file Image_1.JPEG]

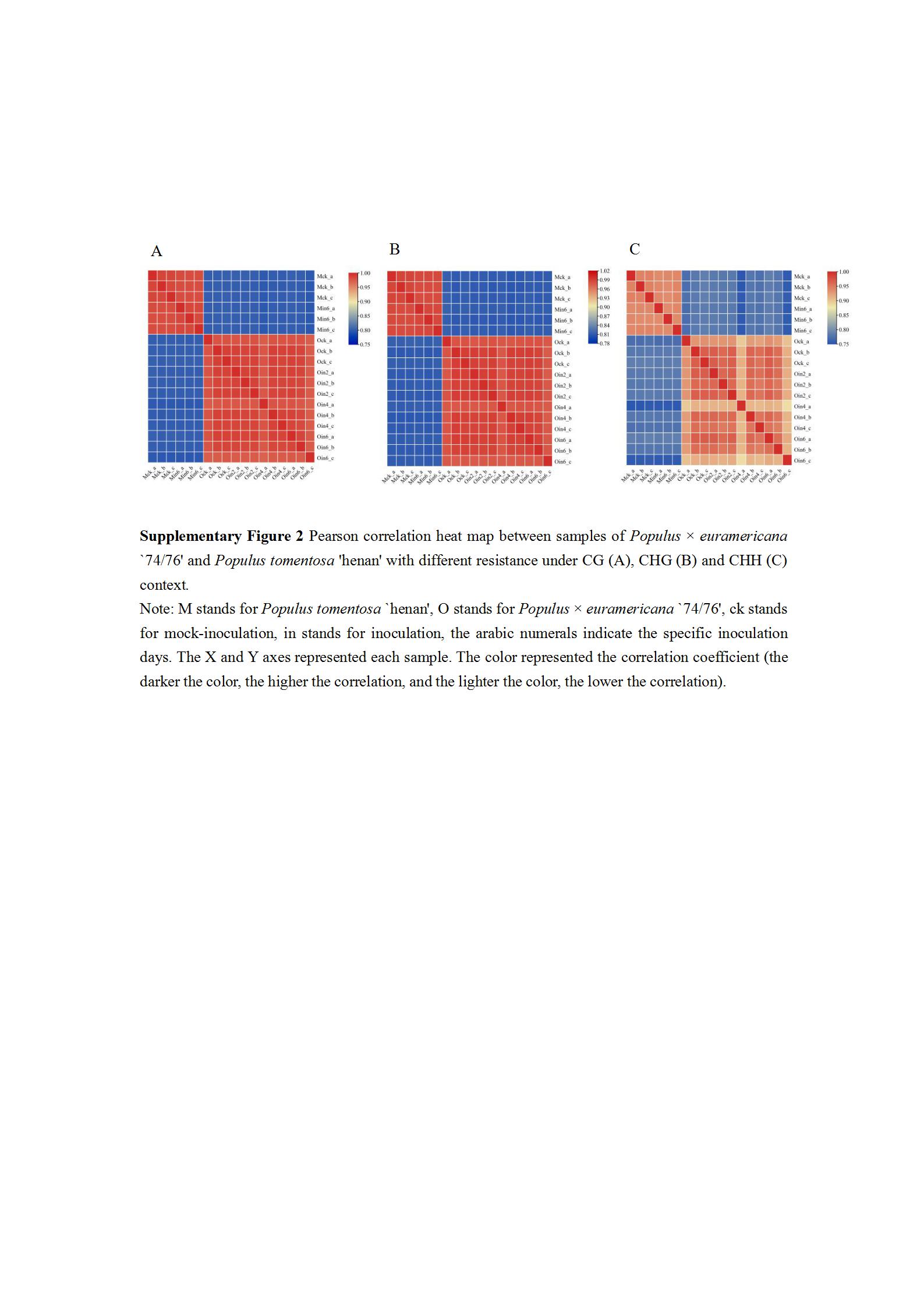

Supplement: Supplementary file 10 [file Image_2.JPEG]

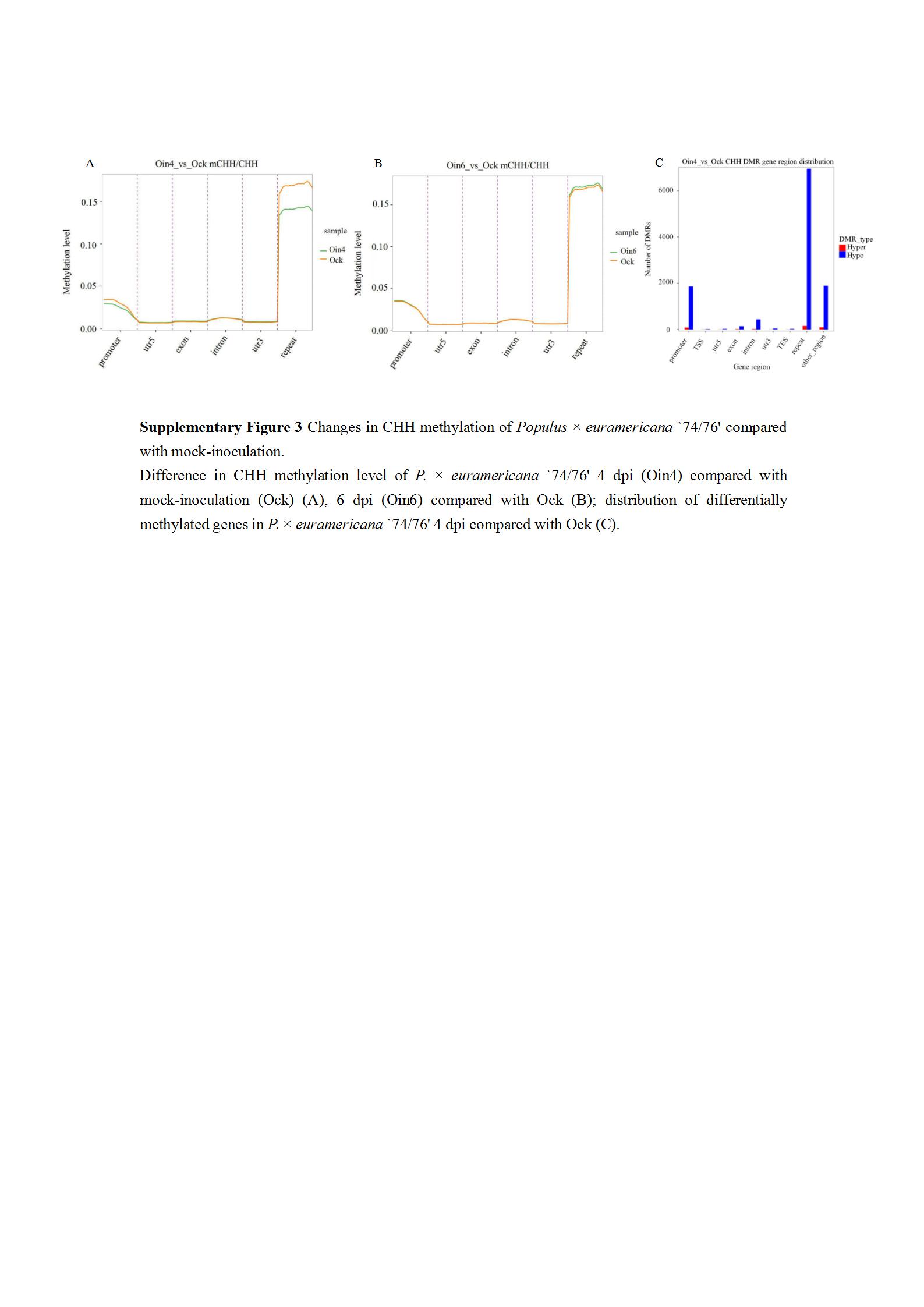

Supplement: Supplementary file 11 [file Image_3.JPEG]

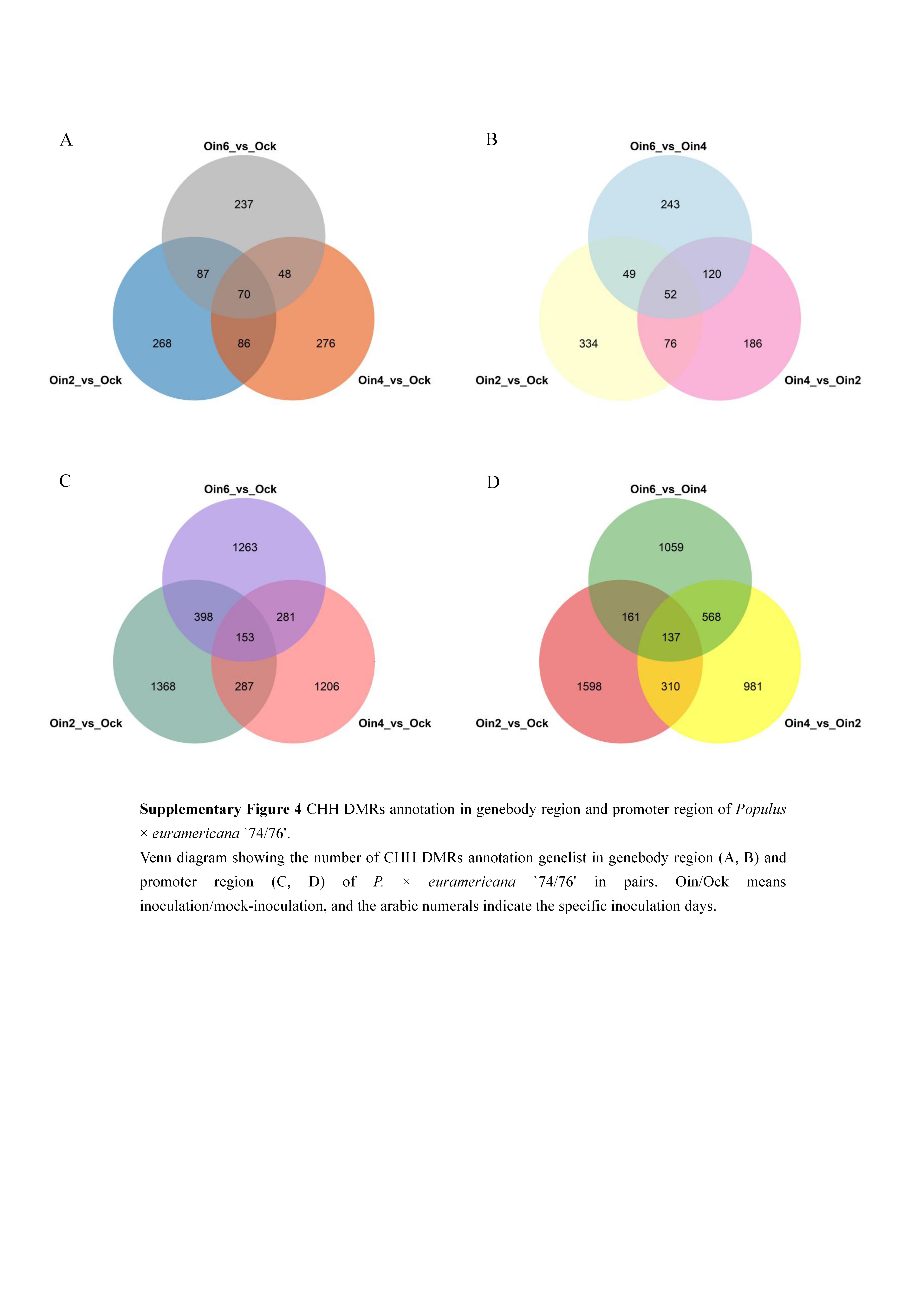

Supplement: Supplementary file 12 [file Image_4.JPEG]

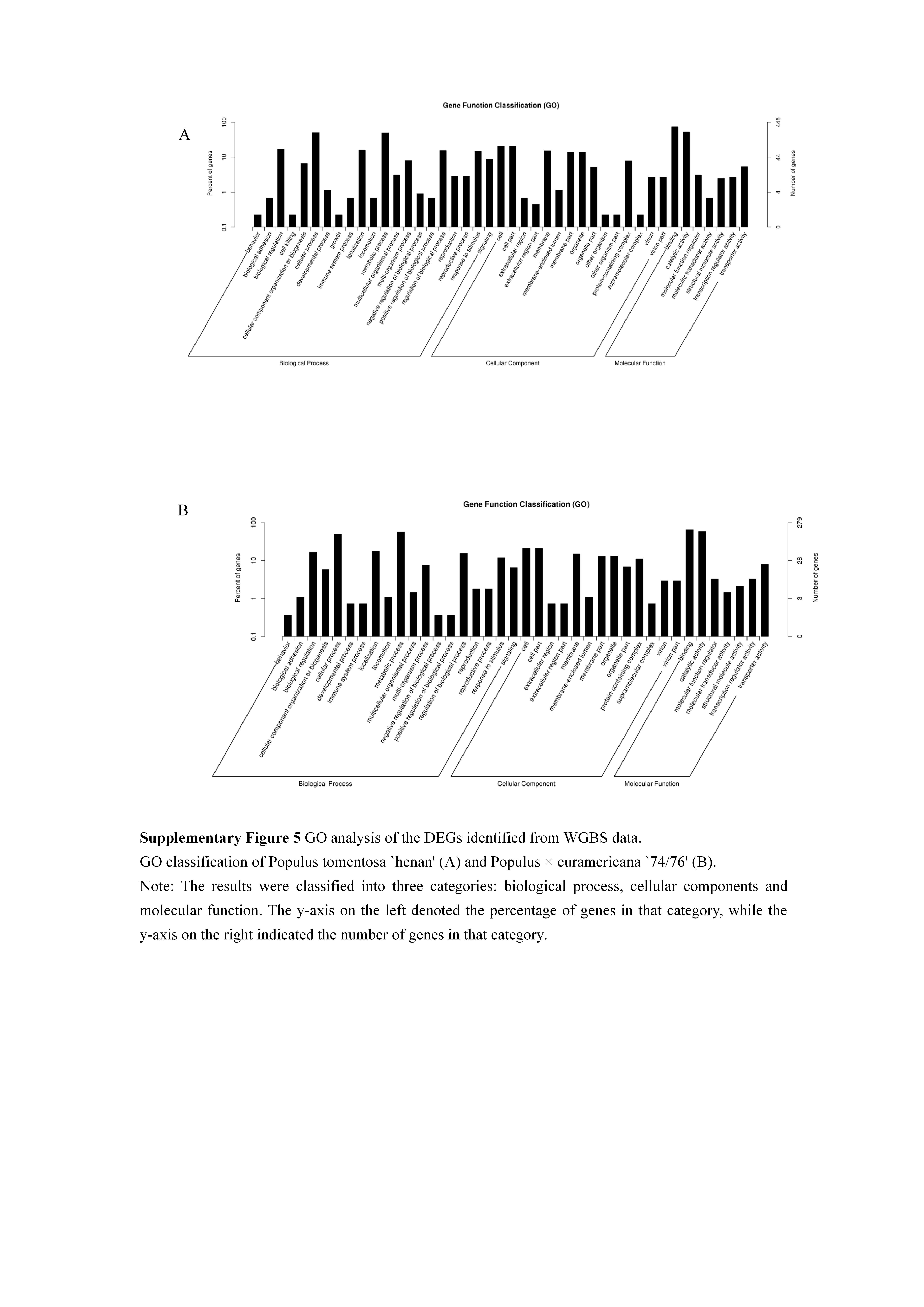

Supplement: Supplementary file 13 [file Image_5.JPEG]

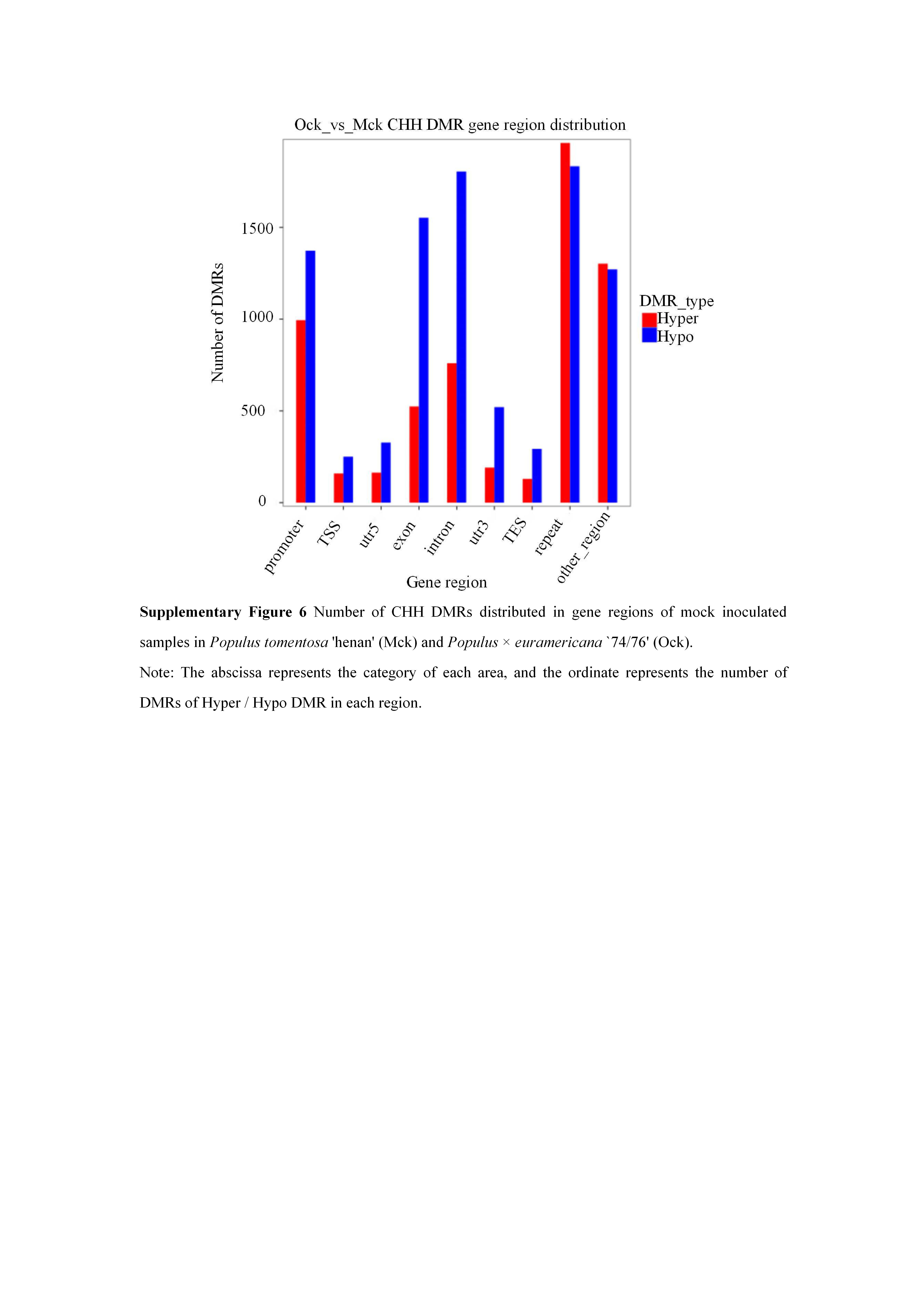

Supplement: Supplementary file 14 [file Image_6.JPEG]

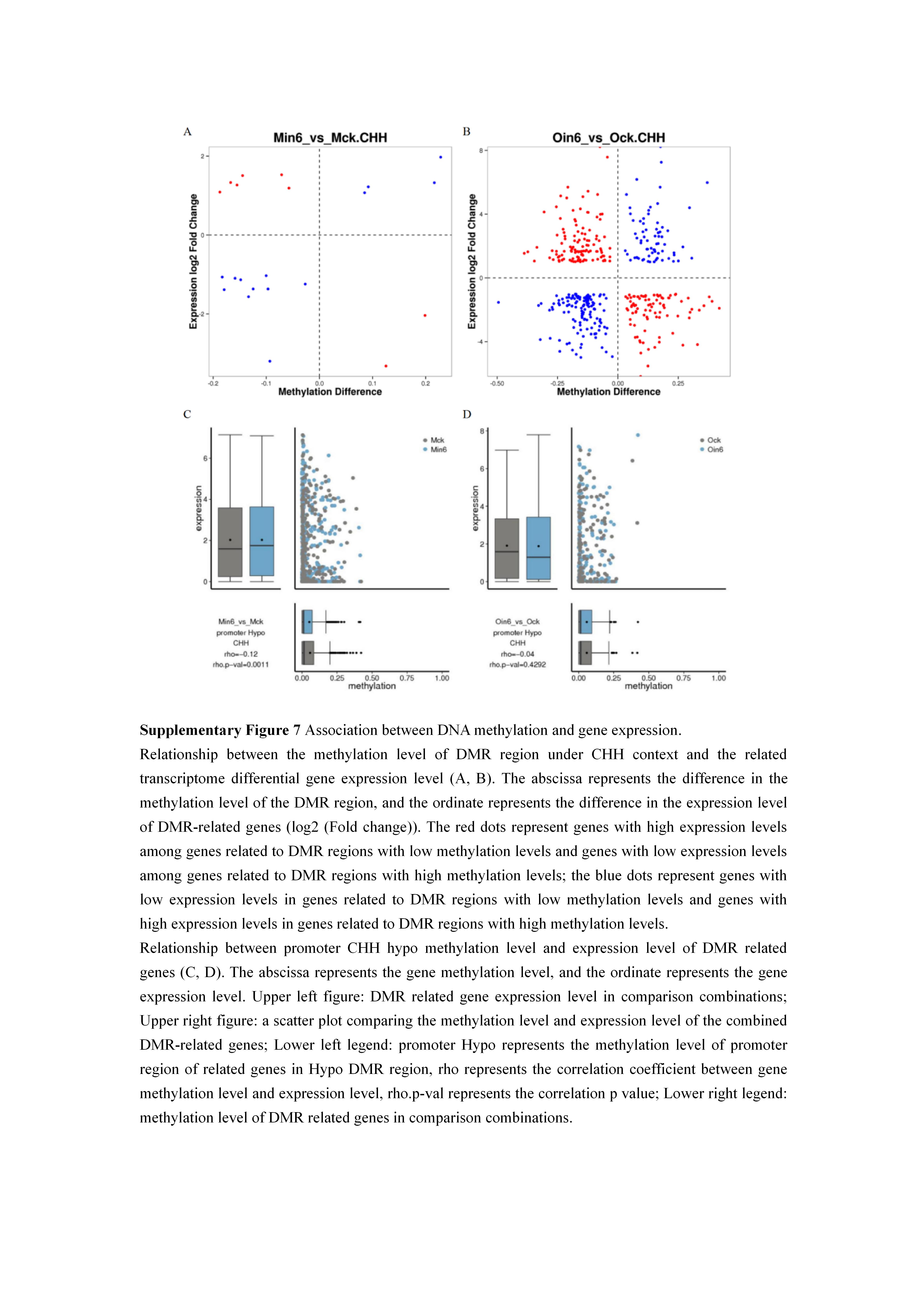

Supplement: Supplementary file 15 [file Image_7.JPEG]

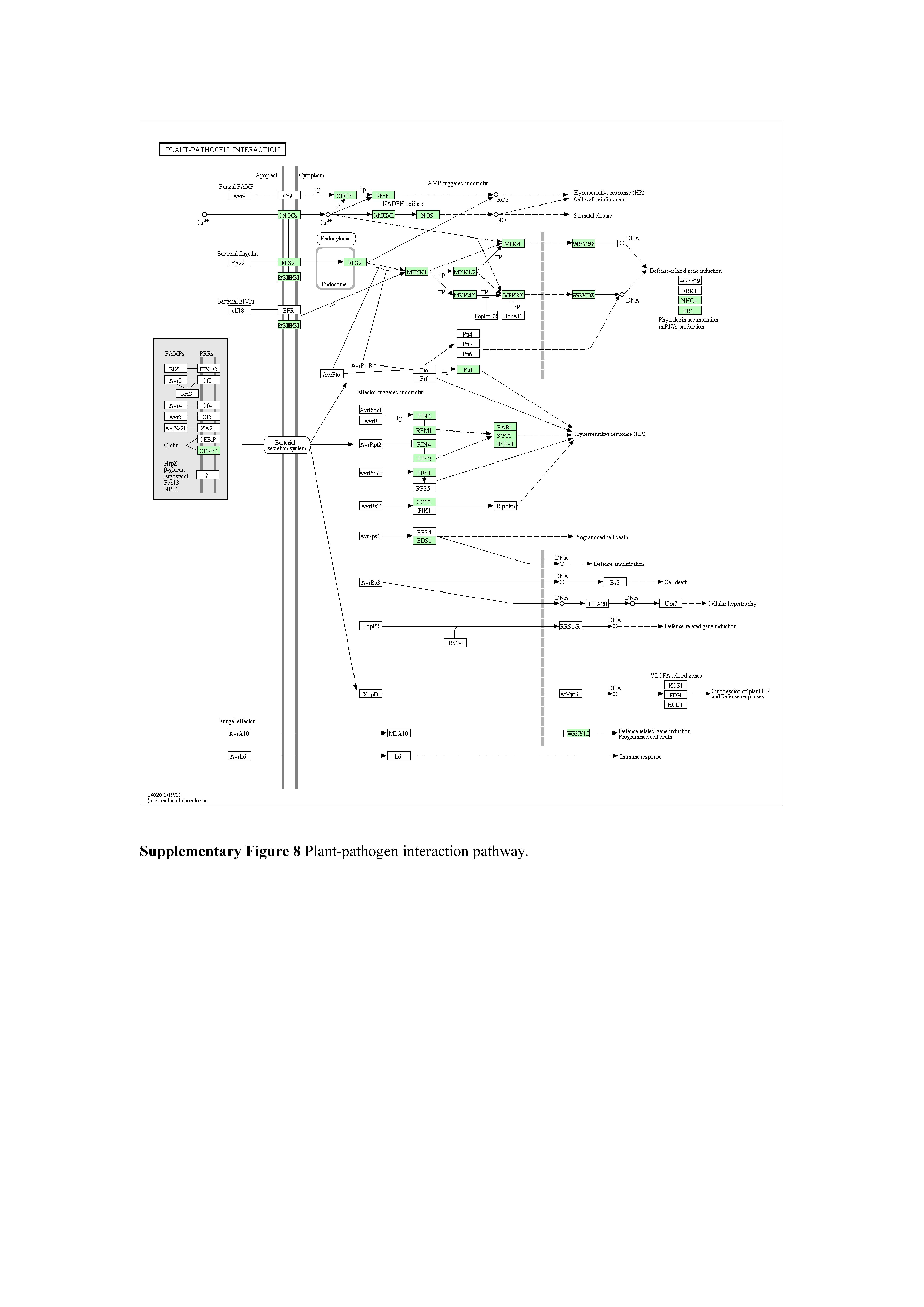

Supplement: Supplementary file 16 [file Image_8.JPEG]

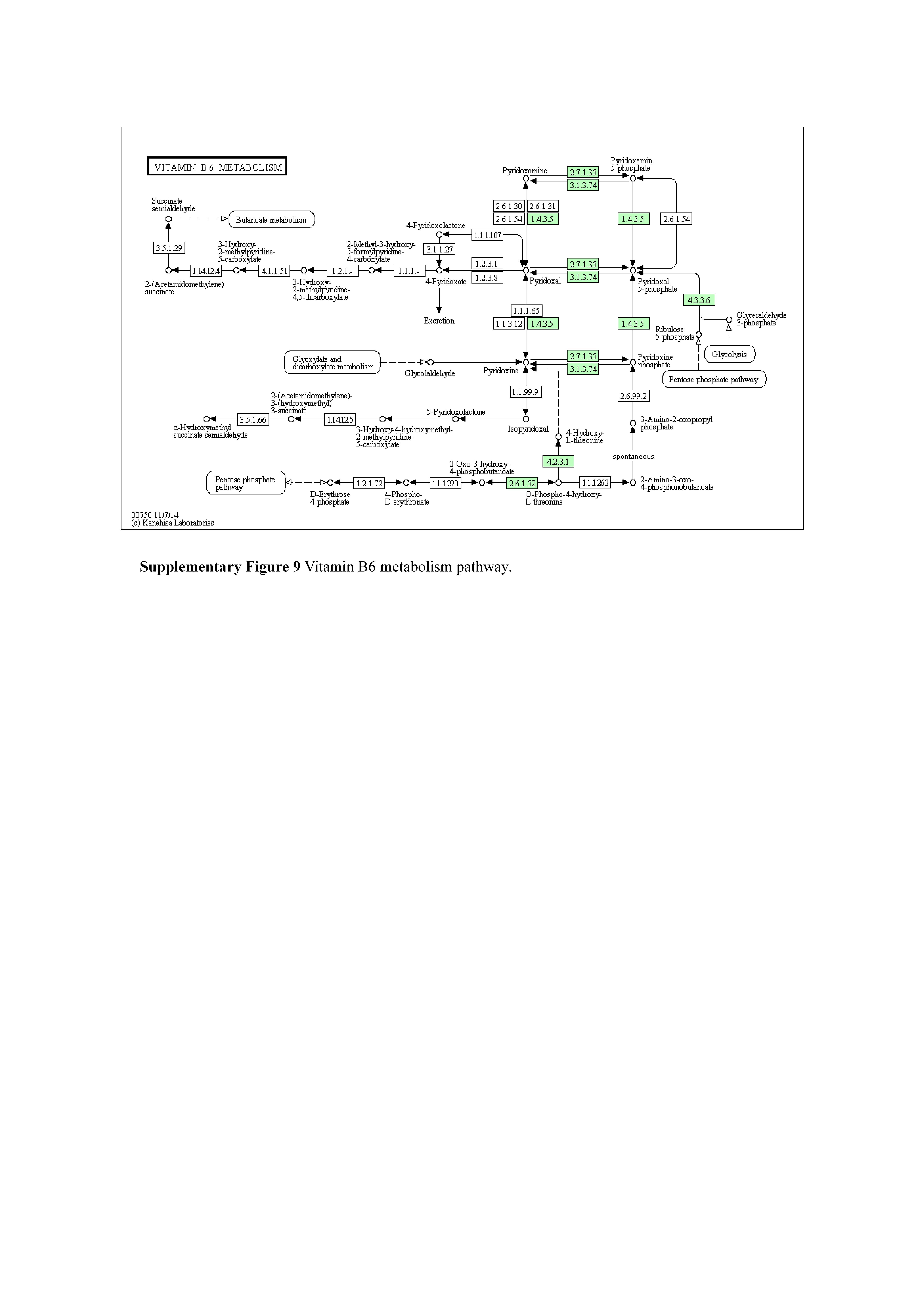

Supplement: Supplementary file 17 [file Image_9.JPEG]

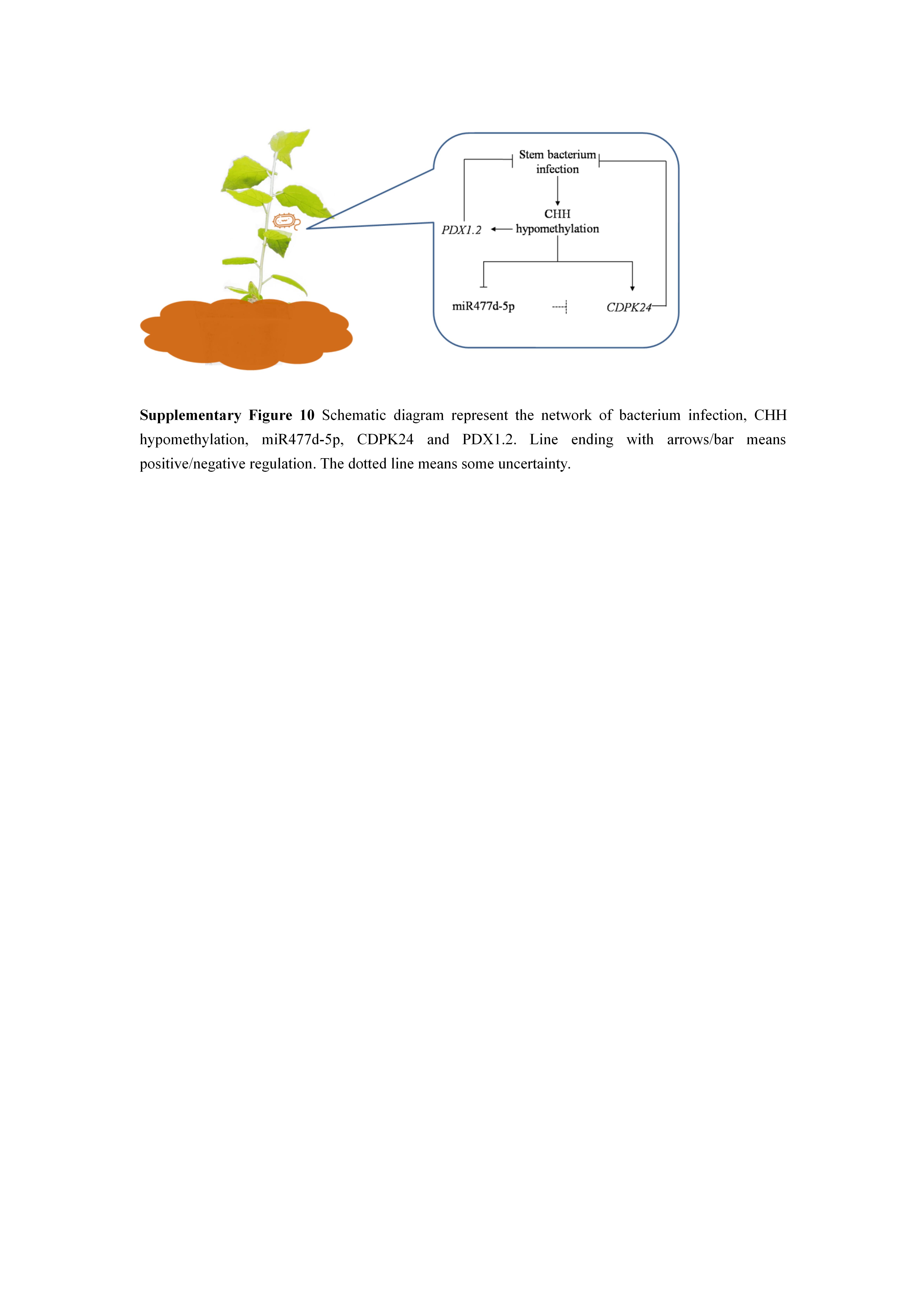

Supplement: Supplementary file 18 [file Image_10.JPEG]
